# Implementation of hand hygiene in health-care facilities: results from the WHO Hand Hygiene Self-Assessment Framework global survey 2019

**DOI:** 10.1016/S1473-3099(21)00618-6

**Published:** 2022-06

**Authors:** Marlieke E A de Kraker, Ermira Tartari, Sara Tomczyk, Anthony Twyman, Laurent C Francioli, Alessandro Cassini, Benedetta Allegranzi, Didier Pittet

**Affiliations:** a Infection Control Programme and WHO Collaborating Centre on Patient Safety, Geneva University Hospitals and Faculty of Medicine, Geneva, Switzerland; b Faculty of Health Sciences, University of Malta, Msida, Malta; c Department for Infectious Disease Epidemiology, Robert Koch Institute, Berlin, Germany; d Infection Prevention and Control Technical and Clinical Hub, Department of Integrated Health Services, WHO, Geneva, Switzerland; e Program in Medical and Population Genetics, Broad Institute of MIT and Harvard, Cambridge, MA, USA

## Abstract

**Background:**

Hand hygiene is at the core of effective infection prevention and control (IPC) programmes. 10 years after the development of the WHO Multimodal Hand Hygiene Improvement Strategy, we aimed to ascertain the level of hand hygiene implementation and its drivers in health-care facilities through a global WHO survey.

**Methods:**

From Jan 16 to Dec 31, 2019, IPC professionals were invited through email and campaigns to complete the online Hand Hygiene Self-Assessment Framework (HHSAF). A geospatial clustering algorithm selected unique health-care facilities responses and post-stratification weighting was applied to improve representativeness. Weighted median HHSAF scores and IQR were reported. Drivers of the HHSAF score were determined through a generalised estimation equation.

**Findings:**

3206 unique responses from 90 countries (46% WHO Member States) were included. The HHSAF score indicated an intermediate hand hygiene implementation level (350 points, IQR 248–430), which was positively associated with country income level and health-care facility funding structure. System Change had the highest score (85 points, IQR 55–100), whereby alcohol-based hand rub at the point of care has become standard practice in many health-care facilities, especially in high-income countries. Institutional Safety Climate had the lowest score (55 points, IQR 35–75). From 2015 to 2019, the median HHSAF score in health-care facilities participating in both HHSAF surveys (n=190) stagnated.

**Interpretation:**

Most health-care facilities had an intermediate level of hand hygiene implementation or higher, for which health-care facility funding and country income level were important drivers. Availability of resources, leadership, and organisational support are key elements to further improve quality of care and provide access to safe care for all.

**Funding:**

WHO, Geneva University Hospitals and Faculty of Medicine, and WHO Collaborating Center on Patient Safety, Geneva, Switzerland.

## Introduction

Health-care-associated infections (HAIs) affect the quality of health-care services, jeopardising patient safety and increasing health-care costs. Up to 2·6 million HAIs occur every year in the EU and European Economic Area (approximately 500 per 100 000 inhabitants), resulting in more than 91 000 deaths.^1^ Similarly, in the USA, 1·7 million HAIs (approximately 520 per 100 000 inhabitants) are reported annually, resulting in about 99 000 deaths.^2^ For regions with a greater number of low-income and middle-income countries, data are scant, but available evidence suggests that the incidence of HAIs is higher, and health and economic consequences more dire.^3^

Improvement in hand hygiene compliance has been highlighted as the most effective measure to reduce transmission of pathogenic microorganisms in health care and lower the incidence of HAI in health-care settings,^4^, ^5^ which is even more relevant in the current COVID-19 pandemic. As such, hand hygiene compliance has become one of the key performance indicators of patient safety and quality of health services worldwide.^6^ Unfortunately, overall, hand hygiene compliance remains insufficient, and compliance levels as low as 9% have been reported for health-care facilities from low-income countries.^7^ Although levels for high-income countries are generally higher, they rarely exceed 70%.^8^

In response to these challenges, WHO has placed strong emphasis on improving hand hygiene practices globally. In 2009, WHO launched the Multimodal Hand Hygiene Improvement Strategy (MMIS) together with the Implementation Toolkit, which includes the Hand Hygiene Self-Assessment Framework (HHSAF),^9^, ^10^ to evaluate the level of implementation of hand hygiene programmes and assess improvements over time. Earlier studies demonstrated the validity of the HHSAF tool^11^ and the effectiveness of MMIS implementation and HHSAF evaluation to improve hand hygiene compliance at health-care facilities in different settings.^5^, ^12^, ^13^, ^14^, ^15^, ^16^, ^17^ Furthermore, two global surveys using the HHSAF, in 2011 and 2015, showed the large variability in hand hygiene implementation between health-care facilities.^14^ Unfortunately, these surveys could not identify underlying drivers, and representativeness was limited, especially for low-income countries.

**Research in context**

**Evidence before this study**

We searched PubMed, *medRxiv, bioRxiv, arXiv*, and WHO Global Health Databases for peer-reviewed articles or preprints in English, French, and Spanish that reported international assessments of hand hygiene programme implementation at the health-care facility level according to the WHO validated Hand Hygiene Self-Assessment Framework, between Jan 1, 2010, and April 30, 2021. The search strategy used the terms “healthcare-associated infection”, “infection prevention”, “hand hygiene”, “multimodal strategy”, “alcohol-based hand rub”, “hand hygiene self-assessment framework”, “organizational culture”, and similar terms. We identified one international report, from WHO, using the Hand Hygiene Self-Assessment Framework, which reported data from surveys in 2011 (2119 health-care facilities, 69 countries) and 2015 (807 health-care facilities, 91 countries), focused on the status of hand hygiene programmes and the progress of hand hygiene implementation over time of only 86 health-care facilities. The study found that 68 of 86 participating health-care facilities had an intermediate or advanced level of hand hygiene implementation, which had increased significantly between 2011 and 2015. System Change was the highest scoring element, while Institutional Safety Climate was the lowest. In addition, a number of reports are available from the regional, national, or health-care facility level, including the following WHO regions; Americas (USA), Europe (Italy and Greece), Eastern Mediterranean (Pakistan), Africa (South Africa), and the Western Pacific (Cambodia, Japan, and Korea). These studies reported an intermediate or advanced level of hand hygiene implementation in health-care facilities of high-income countries, but a basic or inadequate level in health-care facilities of low-income countries. Overall, System Change was the element that scored the highest while Institutional Safety Climate scored the lowest.

**Added value of this study**

To our knowledge, this is the largest global survey (3206 health-care facilities, 90 countries) assessing the characteristics, implementation, and progress of hand hygiene programmes worldwide using a validated tool based on the WHO Multimodal Hand Hygiene Improvement Strategy. Compared with earlier efforts, it includes over a quarter of all low-income and lower-middle income countries and provides a global situation analysis of hand hygiene implementation across all income levels in the six WHO regions (Africa, the Americas, Eastern Mediterranean, Europe, South-East Asia, and the Western Pacific). Moreover, representativeness of results was optimised through rigorous methodology; ministries of health were actively approached to increase response rates, unique responses per health-care facility were selected, a threshold was applied to exclude countries with low (and possibly biased) response rates, and post-stratification weighting was applied for important factors that could influence scores. In addition, multivariable models were applied to identify the most important drivers of hand hygiene implementation. In this 2019 global survey, we observed large hand hygiene implementation differences across country income levels, and across health-care facility's funding structure (private versus public funding); availability of more resources was associated with significantly higher scores. Through a geospatial clustering algorithm, we were also able to identify and compare results of 190 health-care facilities that participated in the survey in 2015 and 2019, showing that the level of hand hygiene implementation has stagnated.

**Implications of all the available evidence**

This study shows that most participating health-care facilities have an intermediate level of hand hygiene implementation or higher, although this was positively associated with country income level and health-care facility funding structure (public *vs* private funding). A quarter of health-care facilities, mostly from low-income countries, have a basic or inadequate level of hand hygiene implementation with lack of resources hampering improvement. Our findings highlight that, in low-income countries, Evaluation and Feedback (34 points, IQR 15–65) is the weakest element of hand hygiene implementation; in these regions immediate and systematic feedback on hand hygiene performance needs strengthening, and alcohol-based handrub consumption needs to increase. In high-income countries, Institutional Safety Climate was still the lowest scoring element, indicating that leadership engagement and assignment of hand hygiene leaders is a complex hurdle. This finding is further underlined by the fact that the level of hand hygiene implementation has stagnated in the 190 health-care facilities participating in the 2015 and 2019 survey. Further improvement is needed if health systems worldwide are to provide safe health care, with the ability to prevent and control outbreaks, particularly in low-income settings and publicly funded health-care facilities. The call for a stronger implementation of effective hand hygiene programmes is urgent, as demonstrated by the COVID-19 pandemic, which underlined how infection prevention and control is our first, and most important line of defence. The WHO Hand Hygiene Self-Assessment Framework is a useful model to set-up, evaluate, and improve the organisation of hand hygiene promotion strategies, as a fundamental aspect of an effective infection prevention and control programme, to reduce pathogen transmission, and provide safe health-care for all.

10 years after the release of the WHO MMIS,^10^ it is key to evaluate its worldwide level of implementation and to identify important drivers and barriers to further improve patient safety. Therefore, the objectives of this work were to determine the hand hygiene implementation level in health-care facilities through the global roll-out of the HHSAF survey tool, to explore critical elements of MMIS, to determine factors associated with the HHSAF score, and to evaluate changes over time by comparing results between the 2019 and 2015 surveys.^14^

## Methods

### Survey instrument

The HHSAF is a self-administered questionnaire designed to obtain a systematic situation analysis of hand hygiene structures, resources, promotion, and practices within a health-care facility, and has been validated to measure hand hygiene implementation level, which has been indirectly linked to HAI rates.^4^, ^9^, ^11^ It consists of 27 indicators (each ten to 50 points), distributed over five MMIS^10^ elements (each 100 points: System Change, Training and Education, Evaluation and Feedback, Reminders in the Workplace, and Institutional Safety Climate), with a maximum overall score of 500 points. Based on the overall score, a hand hygiene level can be assigned to an individual health-care facility: inadequate (0–125 points), basic (126–250), intermediate (251–375), or advanced (376–500; appendix pp 2–10).

The survey was approved by the WHO ethics committee (ERC 0003127). Local ethics was not sought or needed because no personal information was collected.

### Study design and participants recruitment

Between Jan 16 and Dec 31, 2019, WHO implemented a worldwide, cross-sectional survey for health-care facilities, focusing on the HHSAF, as well as the Infection Prevention and Control (IPC) assessment framework.^18^ WHO developed a dedicated, password-protected, online data entry form with internal validity checks, which was piloted and improved by 20 participants from low-income and high-income countries. In the instructions, it was underlined that IPC professionals should complete the surveys, in collaboration with relevant colleagues, and a number of basic questions about the health-care facility and the respondent were included. The survey instrument was available in Chinese, Italian, English, French, German, Japanese, Russian, and Spanish.

Since national health-care facility registries are not systematically available, a convenience sample of health-care facilities was accepted per country. For global representativeness, we aimed for a minimum participation of 25% of WHO Member States from each WHO region, and each World Bank income level, as a balance between improving respresentativeness compared with the previous HHSAF survey (19% participation from low-income countries) and being realistic. Health-care facilities were invited to participate through email sent from the global WHO IPC team to 22 144 IPC contact persons from 182 WHO Member States registered to the SAVE LIVES: Clean Your Hands campaign, and promotion through the WHO website, social media, WHO Global IPC Network, WHO Partnership for Patient Safety, and IPC conferences. WHO Headquarters approached WHO regional and country offices to promote participation at national level. In 38 of 194 WHO Member States, ministries of health expressed interest to implement a nationally coordinated data collection.

### Survey selection process

**Figure 1:**
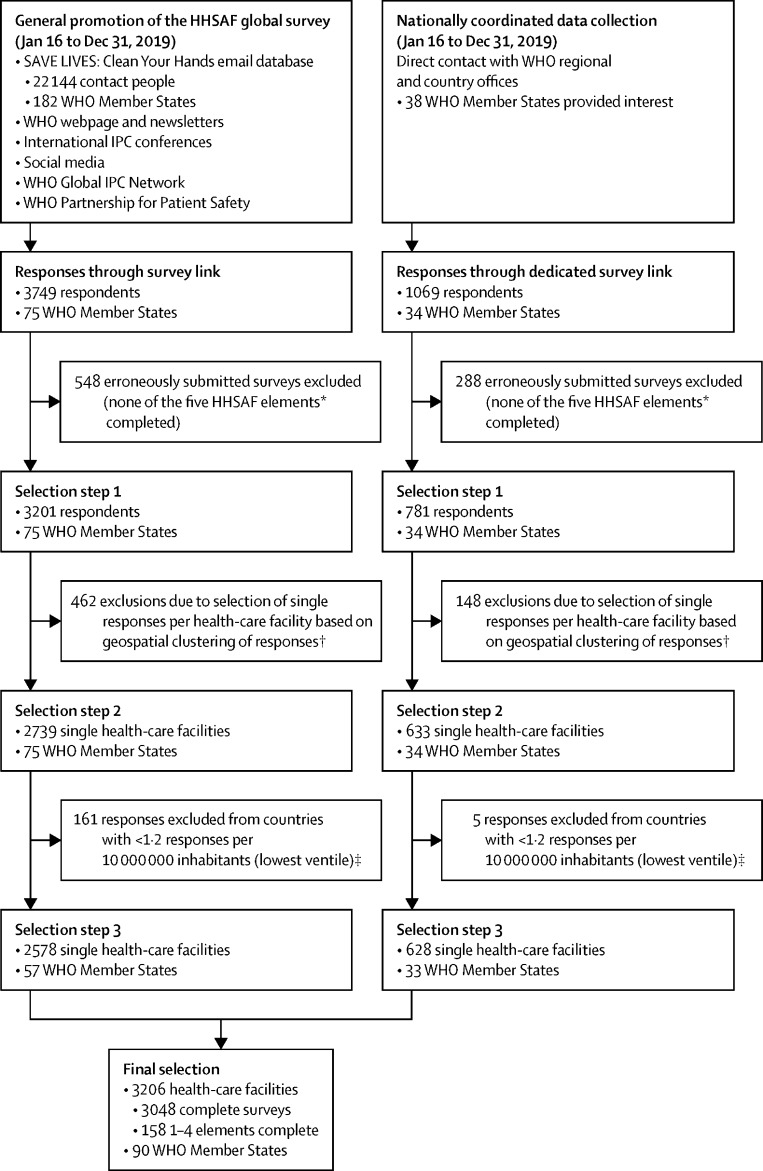
Overview of the selection process for the Hand Hygiene Self-Assessment Framework global survey 2019 with number of selected responses per step HHSAF=Hand Hygiene Self-Assessment Framework. IPC= infection prevention and control. *The HHSAF consists of 27 indicators, distributed over five elements: System Change, Training and Education, Evaluation and Feedback, Reminders in the Workplace, and Institutional Safety Climate. †Participation in the HHSAF global survey was on an individual basis; therefore, multiple survey responses could originate from the same health-care facility. To eliminate duplication, responses from the same health-care facilities were identified through a geospatial clustering algorithm, and a single response per health-care facility was selected through a pre-defined selection strategy. Health-care facility was selected through a predefined selection strategy. ‡The following countries (number of survey responses) were excluded: Togo (n=1), Israel (n=1), Democratic Republic of the Congo (n=9), Hungary (n=1), Ethiopia (n=11), Sri Lanka (n=2), China (n=85), Morocco (n=2), Republic of Korea (n=1), Cameroon (n=1), Kenya (n=2), Brazil (n=8), Venezuela (n=1), USA (n=11), Peru (n=1), Indonesia (n=8), Algeria (n=1), Pakistan (n=5), and India (n=15).

After database lock (April 4, 2020), three selection steps were applied to the received survey responses. In step 1, data from all surveys that fully completed at least one HHSAF element were selected. In step 2, multiple responses per health-care facility were identified through a geospatial clustering algorithm, and a single response per health-care facility was selected using a predefined selection strategy. Two strategies were applied to improve global representativeness: responses from countries with a ratio of number of survey responses per capita in the lowest ventile were excluded from the analysis (selection step 3), and post-stratification weighting was applied through raking, including country, World Bank country income level,^19^ WHO region, facility level (primary, secondary, or tertiary), and private or public health-care facility (figure 1; appendix pp 11–12).

### Statistical analysis

All reported medians are weighted. Descriptive, absolute frequencies and proportions were reported (ie, n in table 1), as well as weighted frequencies and proportions for HHSAF score results (ie, nw). For all analyses related to a specific HHSAF element, all surveys with complete data for that element were included. For analyses related to the overall HHSAF score, only complete surveys were considered.

**Table 1:** Descriptive characteristics of the responses to the Hand Hygiene Self-Assessment Framework (HHSAF) survey 2019

		**All responses (n=3206; %)**
WHO region		
	Africa	649 (20·2%)
	Americas	516 (16·1%)
	Eastern Mediterranean	545 (17·0%)
	Europe	654 (20·4%)
	South East Asia	72 (2·2%)
	Western Pacific	770 (24·0%)
World Bank income level		
	Low income	122 (3·8%)
	Lower-middle income	771 (24·0%)
	Upper-middle income	1230 (38·4%)
	High income	1083 (33·8%)
Nationally coordinated data collection (yes)		628 (19·6%)
Facility type		
	Public	2423 (75·6%)
	Private	662 (20·6%)
	Other*	121 (3·8%)
Facility level of care		
	Primary	1253 (39·1%)
	Secondary	902 (28·1%)
	Tertiary	725 (22·6%
	Other†	326 (10·2%)

Data are n (%).

* This includes mixed private and public facilities, mission hospitals, or unspecified.

† Specialised centres (eg, cardiology, HIV, nephrology, neurology, oncology, obstetrics, plastic surgery, palliative care, rehabilitation, turberculosis).

To determine the independent association between factors considered as strata and the total HHSAF score, a generalised estimating equation (GEE) model was applied with robust standard errors (R package geepack, function geeglm) to accommodate clustering at country level, as well as post-stratification weights. The Box-Cox log-likelihood test was used to assess appropriateness of linear regression. No variable selection procedure was applied, as all available factors were considered relevant.

The previous HHSAF global survey was conducted from June, 2015, to January, 2016 (2015 survey);^14^ it collected data electronically and through paper forms using the same version of the HHSAF, and had a similar distribution method, although it did not include nationally coordinated data collection. Overall, 802 health-care facility responses could be included. Through the geospatial clustering algorithm, health-care facilities that responded to both surveys were identified, and their overall and stratum-specific HHSAF scores were compared using the Wilcoxon signed-rank test. Survey responses from both years were classified according to the World Bank classification from 2015 and 2019.

All analyses were carried out using R, version 3.6.1. p values less than 0·05 were considered statistically significant.

### Role of the funding source

WHO with support from the WHO Collaborating Centre on Patient Safety conceived the study design and carried out data collection, data analysis, data interpretation, and writing of the Article. All authors had full access to the study data and accept responsibility to submit for publication.

## Results

3982 HHSAF survey responses with at least one fully completed survey element were received from 109 countries, 34 of which had a nationally coordinated data collection initiative. After geospatial clustering, 3372 unique health-care facilities were identified. One to 504 responses per country were received, for which the ratio of responses per capita ranged from 4·849 per 100 000 to 0·001 per 100 000 and the lowest ventile cut-off was 0·012 per 100 000. After application of the threshold, 3206 unique responses remained from 90 countries (46% of WHO Member States), 3048 of which were complete surveys, and 158 of which had between one and four elements completed (figure 1).

**Figure 2:**
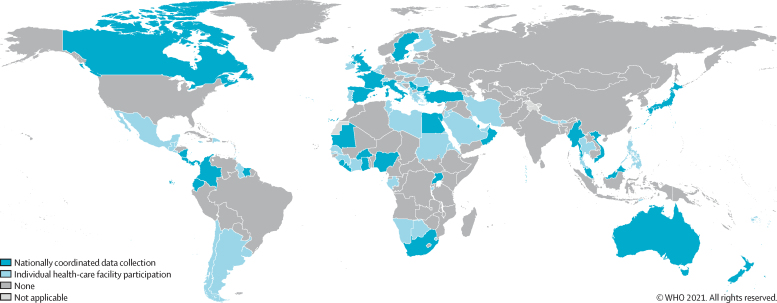
Country origin of survey responses included in the current analysis of the global Hand Hygiene Self-Assessment Framework survey 2019 Total number of countries=90. Total number with nationally coordinated data collection=33. The designations employed and the presentation of the material in this publication do not imply the expression of any opinion whatsoever on the part of WHO concerning the legal status of any country, territory, city or area or of its authorities, or concerning the delimitation of its frontiers or boundaries. Dotted and dashed lines on maps represent approximate border lines for which there may not yet be full agreement. Published with permission of the WHO GIS Centre for Health, DNA/DDI.

The Americas (22 [63%] of 35) and the Eastern Mediterranean region (13 [62%] of 21) had the highest proportions of countries responding, followed by South-East Asia (six [55%] of 11), Europe (22 [42%] of 53), Africa (18 [38%] of 47), and the Western Pacific region (nine [33%] of 27). The proportion of participating countries was highest for upper-middle-income categories (31 [57%] of 54) and high-income categories (34 [55%] of 62). Country participation was eight (28%) of 29 for low-income categories and 17 (35%) of 49 for lower-middle-income categories (figure 2).

The overall, weighted median HHSAF score was 350 points (IQR 248–430), which reflects an intermediate hand hygiene level, ranging from 233 (152–303, basic level) in low-income countries to 395 (315–440, advanced level) in high-income countries (table 2). Overall, most health-care facilities attained an advanced HHSAF level (nw 1254 [41·7%] of 3008), while 976 (32%) had an intermediate level, followed by 555 (18%) with a basic level, and 223 (7%) with an inadequate level.

**Table 2:** Weighted scores for the five elements of the Hand Hygiene Self-Assessment Framework (HHSAF) survey 2019, overall and stratified

		**System Change**	**Training and Education**	**Evaluation and Feedback**	**Reminders in the Workplace**	**Institutional Safety Climate**	**Total score**
Overall		3165; 85 (55–100)	3161; 75 (45–90)	3137; 70 (40–85)	3153; 70 (50–95)	3102; 55 (35–75)	3048; 350 (248–430)
Region							
	Africa	641; 70 (35–95)	644; 60 (35–85)	639; 55 (20–80)	640; 60 (43–90)	632; 55 (30–65)	624; 292 (165–384)
	Eastern Mediterranean	543; 90 (65–100)	536; 80 (60–90)	534; 75 (55–85)	534; 90 (70–100)	531; 65 (40–80)	527; 388 (297–460)
	Europe	649; 100 (80–100)	647; 75 (60–90)	643; 70 (50–85)	646; 70 (58–85)	642; 55 (40–71)	635; 363 (305–413)
	Americas	514; 75 (50–100)	511; 70 (45–90)	504; 70 (50–85)	507; 63 (48–83)	502; 55 (30–80)	498; 330 (245–423)
	South East Asia	72; 80 (45–85)	71; 63 (25–70)	71; 60 (30–85)	71; 63 (22–87)	70; 30 (10–75)	70; 276 (157–411)
	Western Pacific	746; 100 (55–100)	752; 85 (55–100)	746; 80 (55–95)	755; 80 (65–90)	725; 55 (50–80)	694; 390 (303–440)
Income level							
	Low income	119; 45 (30–71)	121; 40 (32–60)	119; 33·8 (15–65)	118; 50 (30–70)	113; 50 (30–59)	112; 233 (152–303)
	Lower-middle income	747; 65 (40–91)	752; 65 (40–85)	747; 60 (20–80)	756; 65 (40–85)	738; 50 (25–65)	708; 295 (184–375)
	Upper-middle income	1220; 85 (55–100)	1221; 75 (45–90)	1211; 75 (50–90)	1216; 77·5 (60–95)	1209; 60 (35–85)	1194; 366 (263–445)
	High income	1079; 100 (85–100)	1067; 85 (65–95)	1060; 75 (55–90)	1063; 75 (60–95)	1042; 65 (45–85)	1034; 395 (315–440)
Nationally coordinated							
	Yes	617; 75 (35–100)	619; 65 (40–90)	616; 55 (30–75)	621; 62·5 (48–85)	600; 40 (25–65)	587; 300 (183–390)
	No	2548; 85 (55–100)	2542; 75 (50–90)	2521; 72·5 (45–85)	2532; 72·5 (50–95)	2502; 60 (35–80)	2461; 354 (255–433)
Facility type							
	Private	655; 100 (80–100)	653; 80 (65–100)	642; 80 (55–90)	646; 80 (63–95)	633; 65 (50–85)	624; 395 (325–450)
	Public	2390; 65 (40–95)	2392; 60 (35–85)	2381; 55 (25–80)	2389; 60 (43–85)	2354; 45 (25–65)	2312; 280 (183–388)
	Other*	120; 80 (55–100)	116; 60·8 (55–80)	114; 80 (60–85)	118; 70 (54–95)	115; 55 (50–80)	112; 331 (287–424)
Facility level							
	Primary	1233; 85 (55–100)	1235; 75 (45–90)	1223; 70 (45–85)	1230; 70 (50–95)	1209; 55 (30–80)	1179; 350 (255–433)
	Secondary	891; 75 (45–100)	895; 60 (35–90)	888; 65 (25–83)	891; 65 (47·5–85)	874; 50 (35–70)	865; 300 (193–398)
	Tertiary	720; 85 (50–100)	718; 75 (50–90)	714; 65 (30–85)	716; 70 (58–98)	708; 60 (30–75)	701; 350 (215–435)
	Other†	321; 100 (75–100)	313; 90 (60–100)	312; 80 (58–85)	316; 82·5 (65–95)	311; 65 (55–80)	303; 415 (323–435)

Data are n; weighted median (IQR). n includes per element all facilities that completed that survey part.

* Includes mixed private and public facilities, mission hospitals, or unspecified.

† Specialised centres (eg, cardiology, HIV, nephrology, neurology, oncology, obstetrics, plastic surgery, palliative care, rehabilitation, tuberculosis).

The multivariable regression analysis confirmed a significant, positive association between total HHSAF score and country income level, with a difference of −137·9 points (95% CI −79·9 to −195·9) between health-care facilities from low-income and high-income countries. For health-care facilities from lower-middle-income countries, this difference was smaller (−75·7 points [–134·6 to −16·7]), and no significant difference was found between health-care facilities from upper-middle-income or high-income countries. Private health-care facilities also scored significantly higher than public health-care facilities (79·6 points [44·0–115·1]; table 3). Similar associations were observed, when including the element-specific scores as outcome of the regression model (appendix pp 13–17).

**Table 3:** Association between the overall score for the Hand Hygiene Self-Assessment Framework (HHSAF) survey 2019 and country and health-care facility characteristics by generalised estimating equation; country was included as cluster and post-stratification weights were applied

	**Coefficient (95% CI)**	**p value**
**World Bank Income level (ref high)**		
Upper-middle income	−20·0 (−69·4 to 29·4)	0·42
Lower-middle income	−75·7 (−134·6 to −16·7)	0·012
Low income	−137·9 (−195·9 to −79·9)	<0·0001
**WHO region (ref Africa)**		
Americas	−11·5 (−74·4 to 51·3)	0·72
Eastern Mediterranean	13·8 (−41·6 to 69·2)	0·63
Europe	−30·1 (−97·8 to 37·7)	0·39
South East Asia	−45·5 (−121·2 to 30·2)	0·24
Western Pacific	10·5 (−55·2 to 76·3)	0·75
**Nationally coordinated data collection (ref no)**		
Yes	−18·4 (−49·5 to 12·8)	0·25
**Facility level (ref primary)**		
Secondary	−33·2 (−85·0 to 18·6)	0·21
Tertiary	0·00 (−51·7 to 51·7)	1·00
Other*	12·8 (−12·9 to 38·5)	0·33
**Type of facility (ref public)**		
Private	79·6 (44·0 to 115·1)	0·0001
Other†	61·9 (18·0 to 105·7)	0·0057

Only includes complete surveys (n=3048).

* This includes mixed private-public facilities, mission hospitals, or unspecified.

† Specialised centres (eg, cardiology, HIV, nephrology, neurology, oncology, obstetrics, plastic surgery, palliative care, rehabilitation, tuberculosis).

For the HHSAF elements, weighted median scores ranged from 85 (IQR 55–100) for System Change to 55 (35–75) for Institutional Safety Climate, whereby the positive association with country income level was reflected as well (table 2).

The weighted score for System Change ranged from 100 (IQR 85–100) in high-income countries to 45 (30–71) in low-income countries (table 2). Large disparities were observed across country income levels with regards to continuous availability of alcohol-based hand rub (ABHR), both facility wide and at each point of care—from 828 (74·8%) of 1107 in high-income countries down to 58 (17%) of 347 in low-income countries. Most health-care facilities in high-income countries had budget available for continuous procurement of hand hygiene supplies (1003 [90·6%] of 1107) compared with only 230 (66%) of 347 in low-income countries (appendix pp 18–24).

Training and Education had a weighted median score of 75 (IQR 45–90), ranging from 85 (65–95) in high-income countries to 40 (32–60) in low-income countries (table 2). A large proportion of health-care facilities (nw 2565 [80·6%] of 3182) reported regular training, although in less than half of the health-care facilities (nw 1531 [48·1%] of 3182) this was mandatory, and similarly only 1362 [42·8%] of 3182 reported having a dedicated budget for training. Almost all private health-care facilities reported regular training (nw 1391 [91·0%] of 1528), and budget was available in almost two thirds of private facilities (nw 943 [61·7%] of 1528), whereas less than a quarter of public health-care facilities (nw 376 [24·6%] of 1526) had a dedicated budget (appendix pp 25–29).

The weighted median score for Evaluation and Feedback was 70 (IQR 40–85), ranging from 75 (55–90) in high-income countries to 34 (15–65) in low-income countries, the lowest scoring element in the low-income stratum (table 2). Hand hygiene compliance was evaluated by direct observation at least every 3 months at 1378 (44·9%) of 3068 health-care facilities. However, 538 (17·5%) of 3068 health-care facilities reported that they never monitored hand hygiene compliance, and this was true for more than a quarter of health-care facilities from low-income countries (nw 99 [30%] of 331) or lower-middle-income countries (nw 186 [27%] of 686), or public health-care facilities (nw 391 [26·1%] of 1500).

The reported hand hygiene compliance rate according to the WHO observation tool was more than 60% in about half of the health-care facilities (nw 1573 [51·3%] of 3068). 2166 (70·6%) of 3068 health-care facilities provide immediate feedback to health-care workers at the end of each observation session; however, just over 60% of health-care facilities provide systematic feedback to health-care workers (nw 1904 [62·1%] of 3068) or facility leadership (nw 1972 [64·%] of 3068). In low-income countries these proportions were almost half those of high-income countries (127 [42·8%] of 331 for systematic feedback and 113 [34%] of 331 for facility leadership in low-income countries *vs* 759 [72·0%] of 1054 systematic feedback and 801 [75·9%] of 1054 for facility leadership in high-income countries). 960 (31·3%) of 3068 health-care facilities reported having the recommended ABHR consumption of at least 20 L per 1000 patient days, with 28 (9%) of 331 health-care facilities in low-income versus 380 (36·1%) of 1054 in high-income countries (appendix pp 30–38).

The weighted median score for Reminders in the Workplace was 70 (IQR 50–95), ranging from 50 (30–70) in low-income countries to 75 (60–95) in high-income countries (table 2). More than half of all 3117 responding health-care facilities displayed posters in all wards explaining indications for hand hygiene (1770 [56·8%]) and correct techniques for hand rubbing (1679 [53·9%]) and hand washing (1770 [56·8%]), which was lower in the 318 facilities in low-income countries (84 [27%] for hand hygiene indications; 86 [27%] for hand rubbing technique; and 28 [9%] for hand washing technique; appendix pp 39–41).

Institutional Safety Climate had the lowest weighted median score of 55 points (IQR 35–75) with no large differences in high-income (65 [45–85]) versus low-income (50 [30–59]) countries. 916 [29·9%] of 3059 health-care facilities had implemented a formalised programme of patient engagement (348 [33·5%] of 1038 in high-income countries *vs* 44 [14%] of 314 in low-income countries). More than half of health-care facilities (1796 [58·7%] of 3059) had established hand hygiene institutional targets (731 [70·4%] of 1038 in high-income countries *vs* 50 [16%] of 314 in low-income countries). Less than 40% of health-care facilities had hand hygiene role models (1111 [36·3%] of 3059) or champions (1196 [39·1%] of 3059). This proportion ranged from 26 (8%) of 314 for hand hygiene role models and 62 (20%) of 314 for hand hygiene champions in low-income countries up to 431 (41·5%) of 1038 for hand hygiene role models and 489 (47·1%) of 1038 for hand hygiene champions in high-income countries. A similar trend was seen for public versus private health-care facilities (appendix pp 42–45).

190 health-care facilities were identified that participated in the HHSAF surveys in 2015 and 2019, with a total median HHSAF score of 435 (IQR 371–470) in 2015 and 430 (385–460) in 2019. This corresponded to a non-significant, paired median difference in total HHSAF score of −1 (−25 to −35). The total HHSAF score increased significantly for high-income countries by 18 points (p=0·026; table 4). A significant increase in score was also found for Institutional Safety Climate (p=0·0027; median difference 5; appendix p 46), because more health-care facilities (n=103) reported an increase than a decrease (n=60), and the median paired difference was 5 points, which was identified as a significant increase through the non-parametric Wilcoxon-signed rank test (table 4; appendix p 46).

**Table 4:** Differences in scores for the HHSAF survey 2019 versus 2015, overall and per stratum

		**N**	**HHSAF score 2015**	**HHSAF score 2019**	HHSAF score 2019–15*	**Comparison p value**†
Overall		190	435 (371 to 470)	430 (385 to 460)	1·2 (−25 to 35)	0·17
WHO region						
	Africa	9	215 (190 to 353)	355 (317·5 to 440)	100 (−10 to 138)	0·074
	Americas	13	372·5 (250 to 420)	385 (302·5 to 440)	15 (−5 to 45)	0·16
	Eastern Mediterranean	10	397·5 (368 to 423)	417·5 (396·2 to 460)	36·2 (−9 to 87)	0·092
	Europe	36	355 (317 to 413)	386·2 (340·6 to 406·9)	19 (−21 to 63)	0·12
	South East Asia	6	432·5 (276 to 490)	435 (320 to 486)	13 (1 to 44)	0·22
	Western Pacific	116	455 (425 to 475)	445 (417 to 470)	−5 (−30 to 25)	0·17
World Bank income level 2019						
	Low income	1	215	318	103	NA
	Lower-middle income	12	276 (209 to 395)	375 (319 to 398)	30 (−12 to 98)	0·13
	Upper-middle income	118	460 (425 to 479)	448 (418 to 470)	−5 (−28 to 25)	0·41
	High income	59	380 (333 to 430)	395 (351 to 430)	18 (û20 to 61)	0·026
World Bank Income level 2015						
	Low income	2	229 (222 to 236)	306 (301 to 312)	78 (65 to 90)	0·50
	Lower-middle income	15	375 (206 to 445)	395 (341 to 455)	20 (−14 to 105)	0·12
	Upper-middle income	115	455 (423 to 475)	445 (416 to 470)	−5 (−29 to 25)	0·42
	High income	58	381 (336 to 430)	396 (358 to 433)	18 (−20 to 56)	0·035

Data are n, median (IQR), or median of score differences (IQR), unless otherwise specified. Only includes complete survey responses from health-care facilities that responded to both surveys (N=190). HHSAF=Hand Hygiene Self-Assessment Framework. NA=not applicable.

* Median of score differences accounts for the pairing of survey responses, whereby the median of the difference will not be equal to subtraction of the overall median scores for 2019 and 2015.

† Wilcoxon signed-rank test for paired data.

## Discussion

10 years after the development and first application of the HHSAF tool based on the WHO MMIS, this global survey provides a snapshot of the hand hygiene implementation level in 3206 health-care facilities from 90 countries. According to the survey, more than half of the health-care facilities have achieved an intermediate hand hygiene level (median 350 points [IQR 248–430]), although this varied widely with country income level and health-care facility funding structure (private *vs* public). About a quarter of health-care facilities, mainly from low-income countries, reported basic or inadequate levels of hand hygiene implementation. The lowest scoring HHSAF element was Institutional Safety Climate, where lack of patient engagement and absence of hand hygiene leaders were identified. For low-income countries, Evaluation and Feedback had the lowest score due to infrequent immediate and systematic feedback of hand hygiene performance, and low or unknown rates of ABHR consumption.

Despite global efforts, no hand hygiene improvement over time could be identified among 190 health-care facilities between 2015 and 2019. However, this might reflect the high quality of health-care facilities participating in both surveys instead of a true trend over time. Most of the health-care facilities participating in both surveys already had an advanced hand hygiene level (score >375) in 2015, indicating that only a selected number of improvements could still increase the score. This is underlined by the fact that the only HHSAF element with a significant increase was Institutional Safety Climate, the lowest scoring element.

Based on the survey, the level of hand hygiene implementation seems highly dependent on available resources; lower scores across all elements were observed for health-care facilities from low-income versus high-income countries, but also from public versus private health-care facilities, with little variation. This included scores for indicators that could be proxies for availability of resources, like a dedicated budget for ABHR, or continuous availability of hand hygiene supplies. Lack of dedicated IPC budget in health-care facilities increases the risk of HAIs. Data from the International Nosocomial Control Consortium (INICC) surveillance programme have also shown that device-associated infection rates were strongly associated with socioeconomic levels of participating countries.^20^, ^21^, ^22^ At the same time, prevention of HAIs could actually save costs,^23^, ^24^ as these are associated with extended length of hospital stay, more expensive antimicrobial treatment, and increasing resistance rates.^20^ Therefore, there is a clear need for investment in IPC, especially in the most resource-limited settings. This need is further underlined by results from the 2020 water, sanitation and hygiene (WASH) global survey, which reported that, globally, one in three health-care facilities do not have adequate facilities to perform hand hygiene at the point of care, and half of health-care facilities in low-income and middle-income countries lack basic hygiene services.^25^

Increasing funds alone will not be sufficient to improve hand hygiene implementation. The HHSAF element with the lowest overall score was the Institutional Safety Climate, among which indicators like hand hygiene champions and role models, and patient engagement are most frequently not fulfilled, especially in low-income countries. For these countries, systematic feedback to leadership, in the element Evaluation and Feedback, also scored low. While this finding might partly be related to resources, it also indicates that leadership engagement, and organisational support are important elements that could further enhance hand hygiene implementation. INICC identified a link between low-income and lack of legally enforceable IPC regulations and absence of hospital accreditation. In line with our findings, private institutions, for example, had stronger accreditation systems, which included funding for IPC programmes.^21^ Collignon and colleagues^26^ also observed a positive association between corruption, lack of rule of law and antimicrobial resistance, another quality-of-care index, emphasising the importance of reliable leadership for adequate health-care delivery. These findings underline the importance of extending and intensifying initiatives to raise awareness among policy makers and facility leadership about the importance of hand hygiene and their role in adequate and sustained implementation. In addition, a broader economic development of resource-poor settings could have an important impact on IPC implementation as well.

The element Evaluation and Feedback for health-care facilities from low-income countries marked the lowest element-specific and stratum-specific score. Similarly, other reports have shown low levels of surveillance and monitoring of IPC-related indicators in low-income countries, including hand hygiene compliance and ABHR consumption.^27^ Insufficient technical expertise (validated hand hygiene observers) for monitoring systems and processes, as well as lack of access to continuous hand hygiene supplies can be potential drivers, underlining the need for more training, health-care resources, and infrastructure. Hand hygiene monitoring with feedback is recommended as a key performance indicator, and is part of core component six of the WHO guidelines for effective IPC programmes.^6^ Our findings highlight the need for better support of health-care facilities in resource-limited settings to implement this recommendation.

Implementation of elements of the WHO MMIS, such as Training and Education, Reminders in the Workplace, and System Change have already reached high levels in the majority of health-care facilities. Indicators like ABHR availability at the point of care, regular hand hygiene training, or posters in wards seem to have become part of routine care in many health-care facilities, especially in high-income countries. These might represent the most easily achievable indicators, because they do not require important organisational changes, and are visible modifications. Elements or indicators with generally lower scores, such as Institutional Safety Climate or patient engagement, depend more on culture change and excellent practice levels throughout the organisation.

Global health security relies on effective IPC, of which hand hygiene is an important element. Globally, a number of initiatives have been launched to support improvement of hand hygiene implementation with a clear focus on local solutions. The WHO Save Lives: Clean Your Hands campaign is annually celebrated on May 5, and provides an opportunity for IPC teams to actively participate and engage leaders and policy makers in hand hygiene promotional activities. In the context of the current COVID-19 global pandemic, there has been an increased focus by WHO and other global partners to support hand hygiene implementation progress worldwide,^28^, ^29^, ^30^ including the Hand Hygiene for All initiative,^28^, ^29^ launched together with UNICEF. There has never been a more crucial time for these efforts to come together to support global health.

Our study has some limitations. It was not possible to apply a random sampling approach because a complete list of health-care facilities is not systematically available at a global, or national level. Therefore, this was a self-selecting survey, risking attracting only those health-care facilities that are motivated to invest in hand hygiene practices, possibly resulting in an overestimation of the global HHSAF score. This possibility is partly corroborated by the finding that 81% of participating health-care facilities reported regular hand hygiene training in their facility. Nevertheless, comparisons across strata and over time would still be valid if the impact of selection bias has the same size and direction. If multiple responses from the same health-care facility were available, a single response per health-care facility was selected using a predefined selection strategy. This selection could have reduced representativeness of the response, but it also prevented double counting. Moreover, averaging of categorical question answers would not have been meaningful, and in most cases a more complete survey response was selected. In addition, the overall response rate, and country participation from certain regions (Western Pacific) and income levels (low-income countries) was low, which could have reduced global representativeness. Although it is difficult to estimate the overall response rate, it could be approximately 20%, taking the number of contacts in the SAVE LIVES: Clean Your Hands database as an approximate basis (assuming not all of these addresses were valid, but there was wider dissemination as well). This percentage is higher than in previous HHSAF surveys^14^ and is close to previously reported ranges for email-based surveys, even though this survey was relatively long, had very specific questions, required input from colleagues, and attempted to reach facilities in a range of different settings worldwide. Moreover, the impact of differential non-response was reduced through weighting the overall results for region and income level, as well as type and level of facility. Finally, any survey is susceptible to a certain degree of social desirability bias, whereby respondents prefer to select the best answer over the true answer.

This global HHSAF survey included data from 3206 health-care facilities in 46% of all WHO Member States worldwide. Representativeness of results was improved by selecting unique health-care facility responses only, and by applying a threshold to exclude countries with low response rates and post-stratification weights. The reported findings are based on a validated tool, providing the possibility of benchmarking hand hygiene implementation over time and place. This global survey shows that most participating health-care facilities have an intermediate or higher level of hand hygiene implementation, but the level is still basic or below in a quarter of health-care facilities, mostly from low-income countries. The Institutional Safety Climate is one of the most difficult areas to implement, and for low-income countries Hand Hygiene Evaluation and Feedback has most room for improvement. Availability of resources, leadership, and organisational support are key elements to further improve quality of care worldwide and provide access to safe care for all.

## Data sharing

The research protocol for this study included a commitment by WHO to restrain from publicly sharing results from individual health-care facilities, or results per country, to improve participation and minimise social desirability bias. Since aggregated results (per WHO region, World Bank income level, hospital type, and hospital level of care) are already available from the tables in the Article and in the appendix, no other data will be shared.

## Declaration of interests

All authors declare no competing interests.

## References

[1] Cassini A, Plachouras D, Eckmanns T (2016). Burden of six healthcare-associated infections on European population health: estimating incidence-based disability-adjusted life years through a population prevalence-based modelling study. PLoS Med.

[2] Magill SS, O'Leary E, Janelle SJ (2018). Changes in prevalence of health care-associated infections in U.S. Hospitals. N Engl J Med.

[3] Allegranzi B, Bagheri Nejad S, Combescure C (2011). Burden of endemic health-care-associated infection in developing countries: systematic review and meta-analysis. Lancet.

[4] Pittet D, Hugonnet S, Harbarth S (2000). Effectiveness of a hospital-wide programme to improve compliance with hand hygiene. Infection Control Programme. Lancet.

[5] Allegranzi B, Gayet-Ageron A, Damani N (2013). Global implementation of WHO's multimodal strategy for improvement of hand hygiene: a quasi-experimental study. Lancet Infect Dis.

[6] WHO (2016). https://www.who.int/infection-prevention/tools/core-components/en/.

[7] Lambe KA, Lydon S, Madden C (2019). Hand hygiene compliance in the ICU: a systematic review. Crit Care Med.

[8] Erasmus V, Daha TJ, Brug H (2010). Systematic review of studies on compliance with hand hygiene guidelines in hospital care. Infect Control Hosp Epidemiol.

[9] WHO (2010). https://www.who.int/gpsc/5may/tools.

[10] WHO (2009). https://www.who.int/gpsc/5may/Guide_to_Implementation.pdf.

[11] Stewardson AJ, Allegranzi B, Perneger TV, Attar H, Pittet D (2013). Testing the WHO Hand Hygiene Self-Assessment Framework for usability and reliability. J Hosp Infect.

[12] Moro ML, Morsillo F, Nascetti S (2017). Determinants of success and sustainability of the WHO multimodal hand hygiene promotion campaign, Italy, 2007–2008 and 2014. Euro Surveill.

[13] Kritsotakis EI, Astrinaki E, Messaritaki A, Gikas A (2018). Implementation of multimodal infection control and hand hygiene strategies in acute-care hospitals in Greece: a cross-sectional benchmarking survey. Am J Infect Control.

[14] Kilpatrick C, Tartari E, Gayet-Ageron A (2018). Global hand hygiene improvement progress: two surveys using the WHO Hand Hygiene Self-Assessment Framework. J Hosp Infect.

[15] Bert F, Giacomelli S, Ceresetti D, Zotti CM (2019). World Health Organization Framework: multimodal hand hygiene strategy in Piedmont (Italy) health care facilities. J Patient Saf.

[16] Luangasanatip N, Hongsuwan M, Limmathurotsakul D (2015). Comparative efficacy of interventions to promote hand hygiene in hospital: systematic review and network meta-analysis. BMJ.

[17] Schweizer ML, Reisinger HS, Ohl M (2014). Searching for an optimal hand hygiene bundle: a meta-analysis. Clin Infect Dis.

[18] WHO (2018). https://www.who.int/infection-prevention/tools/core-components/IPCAF-facility.PDF.

[19] (2019). https://www.acpe-accredit.org/pdf/ISP/WorldBankData-CountryClassifications.pdf.

[20] Rosenthal VD, Maki DG, Graves N (2008). The International Nosocomial Infection Control Consortium (INICC): goals and objectives, description of surveillance methods, and operational activities. Am J Infect Control.

[21] Rosenthal VD, Maki DG, Salomao R (2006). Device-associated nosocomial infections in 55 intensive care units of 8 developing countries. Ann Intern Med.

[22] Rosenthal VD, Guzman S, Pezzotto SM, Crnich CJ (2003). Effect of an infection control program using education and performance feedback on rates of intravascular device-associated bloodstream infections in intensive care units in Argentina. Am J Infect Control.

[23] Graves N, Weinhold D, Tong E (2007). Effect of healthcare-acquired infection on length of hospital stay and cost. Infect Control Hosp Epidemiol.

[24] Chen YC, Sheng WH, Wang JT (2011). Effectiveness and limitations of hand hygiene promotion on decreasing healthcare-associated infections. PLoS One.

[25] WHO (2020). https://www.who.int/publications/i/item/9789240017542.

[26] Collignon P, Beggs JJ, Walsh TR, Gandra S, Laxminarayan R (2018). Anthropological and socioeconomic factors contributing to global antimicrobial resistance: a univariate and multivariable analysis. Lancet Planet Health.

[27] Tartari E, Tomczyk S, Pires D (2021). Implementation of the infection prevention and control core components at the national level: a global situational analysis. J Hosp Infect.

[28] WHO (2020). https://www.who.int/water_sanitation_health/publications/200831-unicef-hand-hygiene.pdf?ua=1.

[29] WHO (2020). https://www.who.int/publications/i/item/9789240011618.

[30] WHO (2020). https://www.who.int/publications/i/item/recommendations-to-member-states-to-improve-hand-hygiene-practices-to-help-prevent-the-transmission-of-the-covid-19-virus.

