# Supplementary material for: Implementation of hand hygiene in health-care facilities: results from the WHO Hand Hygiene Self-Assessment Framework global survey 2019
Source: Lancet Infect Dis. 2022 Jun;22(6):835–44. doi: 10.1016/S1473-3099(21)00618-6 (PMC9132778; doi:10.1016/S1473-3099(21)00618-6)
Supplement: Supplementary appendix [file mmc1.pdf]

THE LANCET
Infectious Diseases

Supplementary appendix

This appendix formed part of the original submission and has been peer reviewed. We post it as supplied by the authors.

Supplement to: Tomczyk S, Twyman A, de Kraker MEA, et al. The first WHO global survey on infection prevention and control in health-care facilities. *Lancet Infect Dis* 2022; published online February 21. https://doi.org/10.1016/S1473-3099(21)00809-4.

Supplementary appendix

Supplement to: De Kraker et al. Hand Hygiene implementation strongly associated with availability of funds and country income level: Results from the WHO Hand Hygiene Self-Assessment Framework global survey 2019.

Content

1. **Supplementary File S1: WHO Hand Hygiene Self-Assessment Framework**
2. **Supplementary File S2: Survey selection and weighting procedure**
3. **Supplementary File S3: Generalized Estimating Equations (GEE) showing the independent association between element-specific scores for the Hand Hygiene Self-Assessment Framework (HHSAF) survey 2019 and country and healthcare facility characteristics.**
    a. Supplementary Table S3.1: GEE for System Change (N=3165)
    b. Supplementary Table S3.2: GEE for Training and Education (N=3161)
    c. Supplementary Table S3.3: GEE for Evaluation and Feedback (N=3137)
    d. Supplementary Table S3.4: GEE for Reminders in the Workplace (N=3153)
    e. Supplementary Table S3.5: GEE for Institutional Safety Climate (N=3102)
4. **Supplementary File S4: Indicator responses for the Hand Hygiene Self-Assessment Framework (HHSAF) survey 2019 specified per element, weighted for country, country income level, region, facility type and level**
    a. Supplementary Table S4.1: System Change
    b. Supplementary Table S4.2: Training and Education
    c. Supplementary Table S4.3a: Evaluation and Feedback – part I
    d. Supplementary Table S4.3b: Evaluation and Feedback – part II
    e. Supplementary Table S4.4: Reminders in the Workplace
    f. Supplementary Table S4.5a: Institutional Safety Climate – part I
    g. Supplementary Table S4.5b: Institutional Safety Climate – part II
5. **Supplementary File S5: Differences in element-specific scores for the Hand Hygiene Self-Assessment Framework (HHSAF) survey 2019 versus 2015**

Hand Hygiene Self-Assessment Framework 2010

Introduction and user instructions

The Hand Hygiene Self-Assessment Framework is a systematic tool with which to obtain a situation analysis of hand hygiene promotion and practices within an individual health-care facility.

What is its purpose?

While providing an opportunity to reflect on existing resources and achievements, the Hand Hygiene Self-Assessment Framework also helps to focus on future plans and challenges. In particular, it acts as a diagnostic tool, identifying key issues requiring attention and improvement. The results can be used to facilitate development of an action plan for the facility's hand hygiene promotion programme. Repeated use of the Hand Hygiene Self-Assessment Framework will also allow documentation of progress with time.

Overall, this tool should be a catalyst for implementing and sustaining a comprehensive hand hygiene programme within a health-care facility.

Who should use the Hand Hygiene Self-Assessment Framework?

This tool should be used by professionals in charge of implementing a strategy to improve hand hygiene within a health-care facility. If no strategy is being implemented yet, then it can also be used by professionals in charge of infection control or senior managers at the facility directorate. The framework can be used globally, by health-care facilities at any level of progress as far as hand hygiene promotion is concerned.

How is it structured?

The Hand Hygiene Self-Assessment Framework is divided into five components and 27 indicators. The five components reflect the five elements of the WHO Multimodal Hand Hygiene Improvement Strategy (http://www.who.int/gpsc/5may/tools/en/index.html) and the indicators have been selected to represent the key elements of each component. These indicators are based on evidence and expert consensus and have been framed as questions with defined answers (either "Yes/No" or multiple options) to facilitate self-assessment. Based on the score achieved for the five components, the facility is assigned to one of four levels of hand hygiene promotion and practice: Inadequate, Basic, Intermediate and Advanced.

Inadequate: hand hygiene practices and hand hygiene promotion are deficient. Significant improvement is required.

Basic: some measures are in place, but not to a satisfactory standard. Further improvement is required.

Intermediate: an appropriate hand hygiene promotion strategy is in place and hand hygiene practices have improved. It is now crucial to develop long-term plans to ensure that improvement is sustained and progresses.

Advanced: hand hygiene promotion and optimal hand hygiene practices have been sustained and/or improved, helping to embed a culture of safety in the health-care setting.

Leadership criteria have also been identified to recognise facilities that are considered a reference centre and contribute to the promotion of hand hygiene through research, innovation and information sharing. The assessment according to leadership criteria should only be undertaken by facilities having reached the Advanced level.

How does it work?

While completing each component of the Hand Hygiene Self-Assessment Framework, you should circle or highlight the answer appropriate to your facility for each question. Each answer is associated with a score. After completing a component, add up the scores for the answers you have selected to give a subtotal for that component. During the interpretation process these subtotals are then added up to calculate the overall score to identify the hand hygiene level to which your health-care facility is assigned.

The assessment should not take more than 30 minutes, provided that the information is easily available.

Within the Framework you will find a column called "WHO implementation tools" listing the tools made available from the WHO First Global Patient Safety Challenge to facilitate the implementation of the WHO Multimodal Hand Hygiene Improvement Strategy (http://www.who.int/gpsc/5may/tools/en/index.html). These tools are listed in relation to the relevant indicators included in the Framework and may be useful when developing an action plan to address areas identified as needing improvement.

Is the Hand Hygiene Self-Assessment Framework suitable for inter-facility comparison?

Health-care facilities or national bodies may consider adopting this tool for external comparison or benchmarking. However, this was not a primary aim during the development of this tool. In particular, we would draw attention to the risks inherent in using a self-reported evaluation tool for external benchmarking and also advise the use of caution if comparing facilities of different sizes and complexity, in different socioeconomic settings. It would be essential to consider these limitations if inter-facility comparison is to be undertaken.

All reasonable precautions have been taken by the World Health Organization to verify the information contained in this document. However, the published material is being distributed without warranty of any kind, either expressed or implied. The responsibility for the interpretation and use of the material lies with the reader. In no event shall the World Health Organization be liable for damages arising from its use.
WHO acknowledges the Hôpitaux Universitaires de Genève (HUG), in particular the members of the Infection Control Programme, for their active participation in developing this material.

Hand Hygiene Self-Assessment Framework 2010

1. System Change

Question	Answer	Score	WHO improvement tools
**1.1** How easily available is alcohol-based handrub in your health-care facility? **Choose one answer**	Not available	0	→ Ward Infrastructure Survey<br>→ Protocol for Evaluation of Tolerability and Acceptability of Alcohol-based Handrub in Use or Planned to be Introduced:Method 1<br>→ Guide to Implementation II.1
	Available, but efficacy[1] and tolerability[2] have not been proven	0	
	Available only in some wards or in discontinuous supply (with efficacy[1] and tolerability[2] proven)	5	
	Available facility-wide with continuous supply (with efficacy[1] and tolerability[2] proven)	10	
	Available facility-wide with continuous supply, and at the point of care[3] in the majority of wards (with efficacy[1] and tolerability[2] proven)	30	
	Available facility-wide with continuous supply at each point of care[3] (with efficacy[1] and tolerability[2] proven)	50	
**1.2** What is the sink:bed ratio? **Choose one answer**	Less than 1:10	0	→ Ward Infrastructure Survey<br>→ Guide to Implementation II.1
	At least 1:10 in most wards	5	
	At least 1:10 facility-wide and 1:1 in isolation rooms and in intensive care units	10	
**1.3** Is there a continuous supply of clean, running water[4]?	No	0	→ Ward Infrastructure Survey<br>→ Guide to Implementation II.1
	Yes	10	
**1.4** Is soap[5] available at each sink?	No	0	→ Ward Infrastructure Survey<br>→ Guide to Implementation II.1
	Yes	10	
**1.5** Are single-use towels available at each sink?	No	0	→ Ward Infrastructure Survey<br>→ Guide to Implementation II.1
	Yes	10	
**1.6** Is there dedicated/available budget for the continuous procurement of hand hygiene products (e.g. alcohol-based handrubs)?	No	0	→ Guide to Implementation II.1
	Yes	10	

Extra Question: Action plan

Question	Answer	Score	WHO improvement tools
**Answer this question ONLY if you scored less than 100 for questions 1.1 to 1.6:** Is there realistic plan in place to improve the infrastructure[6] in your health-care facility?	No	0	→ Alcohol-based Handrub Planning and Costing Tool<br>→ Guide to Local Production: WHO-recommended Handrub Formulations<br>→ Guide to Implementation II.1
	Yes	5	
	**System Change subtotal**	/100	

1. **Efficacy:** The alcohol-based handrub product used should meet recognised standards of antimicrobial efficacy for hand antisepsis (ASTM or EN standards). Alcohol-based handrubs with optimal antimicrobial efficacy usually contain 75 to 85% ethanol, isopropanol, or n-propanol, or a combination of these products. The WHO-recommended formulations contain either 75% v/v isopropanol, or 80% v/v ethanol.

2. **Skin tolerability:** The alcohol-based handrub product is well tolerated by health-care workers skin (i.e. it does not harm or irritate the skin) when used in clinical care, as demonstrated by reliable data. The WHO Protocol for Evaluation of Tolerability and Acceptability of Alcohol-based Handrub in Use or Planned to be Introduced can be used as a reference.

3. **Point of care:** The place where three elements come together: the patient, the health-care worker, and care or treatment involving contact with the patient or his/her surroundings (within the patient zone). Point-of-care products should be accessible without having to leave the patient zone (ideally within arms reach of the health-care worker or within 2 meters).

4. **Clean, running water:** A water supply that is either piped in (or where this is not available, from onsite storage with appropriate disinfection) that meets appropriate safety standards for microbial and chemical contamination. Further details can be found in Essential environmental health standards in health care (Geneva, World Health Organization, 2008, http://whqlibdoc.who.int/publications/2008/9789241547239_eng.pdf).

5. **Soap:** Detergent-based products that contain no added antimicrobial agents, or may contain these solely as preservatives. They are available in various forms including bar soap, tissue, leaf, and liquid preparations.

6. **Infrastructure:** The "infrastructure" here referred to includes facilities, equipment, and products that are required to achieve optimal hand hygiene practices within the facility. Specifically, it refers to the indicators included in questions 1.1-1.5 and detailed in the WHO Guidelines on Hand Hygiene in Health Care 2009, Part I, Chapter 23.5 (e.g. availability of alcohol based handrub at all points of care, a continuous supply of clean, running water and a sink:bed ratio of at least 1:10, with soap and single-use towels at each sink).

All reasonable precautions have been taken by the World Health Organization to verify the information contained in this document. However, the published material is being distributed without warranty of any kind, either expressed or implied. The responsibility for the interpretation and use of the material lies with the reader. In no event shall the World Health Organization be liable for damages arising from its use.
WHO acknowledges the Hôpitaux Universitaires de Genève (HUG), in particular the members of the Infection Control Programme, for their active participation in developing this material.

Hand Hygiene Self-Assessment Framework 2010

2. Training and Education

Question	Answer	Score	WHO improvement tools
**2.1** Regarding training of health-care workers in your facility:			
2.1a How frequently do health-care workers receive training regarding hand hygiene[7] in your facility? **Choose one answer**	Never	0	→ Slides for Education Session for Trainers, Observers and Health-care Workers → Hand Hygiene Training Films → Slides Accompanying the Training Films → Slides for the Hand Hygiene Co-ordinator → Hand Hygiene Technical Reference Manual → Hand Hygiene Why, How and When Brochure → Guide to Implementation II.2
	At least once	5	
	Regular training for medical and nursing staff, or all professional categories (at least annually)	10	
	Mandatory training for all professional categories at commencement of employment, then ongoing regular training (at least annually)	20	
2.1b Is a process in place to confirm that all health-care workers complete this training?	No	0	
	Yes	20	
**2.2** Are the following WHO documents (available at www.who.int/gpsc/5may/tools), or similar local adaptations, easily available to all health-care workers?			→ Guide to Implementation II.2
2.2a The 'WHO Guidelines on Hand Hygiene in Health-care: A Summary'	No	0	→ WHO Guidelines on Hand Hygiene in Health Care: A Summary
	Yes	5	
2.2b The WHO 'Hand Hygiene Technical Reference Manual'	No	0	→ Hand Hygiene Technical Reference Manual
	Yes	5	
2.2c The WHO 'Hand Hygiene: Why, How and When' Brochure	No	0	→ Hand Hygiene Why, How and When Brochure
	Yes	5	
2.2d The WHO 'Glove Use Information' Leaflet	No	0	→ Glove Use Information Leaflet
	Yes	5	
**2.3** Is a professional with adequate skills[8] to serve as trainer for hand hygiene educational programmes active within the health-care facility?	No	0	→ WHO Guidelines on Hand Hygiene in Health Care → Hand Hygiene Technical Reference Manual → Hand Hygiene Training Films → Slides Accompanying the Training Films → Guide to Implementation II.2
	Yes	15	
**2.4** Is a system in place for training and validation of hand hygiene compliance observers?	No	0	
	Yes	15	
**2.5** Is there is a dedicated budget that allows for hand hygiene training?	No	0	→ Template Letter to Advocate Hand Hygiene to Managers → Template Letter to communicate Hand Hygiene Initiatives to Managers → Template Action Plan → Guide to Implementation II.2 and III.1 (page 33)
	Yes	10	
	**Training and Education subtotal**	**/100**	

7. **Training in hand hygiene:** This training can be done using different methods but the information conveyed should be based on the WHO multimodal hand hygiene improvement strategy or similar material. Training should include the following:
- The definition, impact and burden of health care-associated infection (HCAI)
- Major patterns of transmission of health care-associated pathogens
- Prevention of HCAI and the critical role of hand hygiene
- Indications for hand hygiene (based on the WHO 'My 5 Moments for Hand Hygiene' approach)
- Correct technique for hand hygiene (refer to 'How to Handrub' and 'How to Hand Wash')

8. **A professional with adequate skills:** Medical staff or nursing staff trained in Infection Control or Infectious Diseases, whose tasks formally include dedicated time for staff training. In some settings, this could also be medical or nursing staff involved in clinical work, with dedicated time to acquire thorough knowledge of the evidence for and correct practice of hand hygiene (the minimum required knowledge can be found in the WHO Guidelines on Hand Hygiene in Health Care and the Hand Hygiene Technical Reference Manual).

All reasonable precautions have been taken by the World Health Organization to verify the information contained in this document. However, the published material is being distributed without warranty of any kind, either expressed or implied. The responsibility for the interpretation and use of the material lies with the reader. In no event shall the World Health Organization be liable for damages arising from its use.
WHO acknowledges the Hôpitaux Universitaires de Genève (HUG), in particular the members of the Infection Control Programme, for their active participation in developing this material.

3. Evaluation and Feedback

Question	Answer	Score	WHO improvement tools
**3.1** Are regular (at least annual) ward-based audits undertaken to assess the availability of handrub, soap, single use towels and other hand hygiene resources?	No	0	→ Ward Infrastructure Survey<br>→ Guide to Implementation II.3
	Yes	10	
**3.2** Is health care worker knowledge of the following topics assessed at least annually (e.g. after education sessions)?			
3.2a. The indications for hand hygiene	No	0	→ Hand Hygiene Knowledge Questionnaire for Health-Care Workers<br>→ Guide to Implementation II.3
	Yes	5	
3.2b. The correct technique for hand hygiene	No	0	
	Yes	5	
**3.3 Indirect Monitoring of Hand Hygiene Compliance**			
3.3a Is consumption of alcohol-based handrub monitored regularly (at least every 3 months)?	No	0	→ Soap/Handrub Consumption Survey<br>→ Guide to Implementation II.3
	Yes	5	
3.3b Is consumption of soap monitored regularly (at least every 3 months)?	No	0	
	Yes	5	
3.3c Is alcohol based handrub consumption at least 20L per 1000 patient-days?	No (or not measured)	0	
	Yes	5	
**3.4 Direct Monitoring of Hand Hygiene Compliance** Only complete section 3.4 if hand hygiene compliance observers in your facility have been trained and validated and utilise the WHO 'My 5 Moments for Hand Hygiene' (or similar) methodology			
3.4a How frequently is direct observation of hand hygiene compliance performed using the WHO Hand Hygiene Observation tool (or similar technique)? **Choose one answer**	Never	0	→ WHO Hand Hygiene Observation form<br>→ Hand Hygiene Technical Reference Manual<br>→ Guide to Implementation II.3
	Irregularly	5	
	Annually	10	
	Every 3 months or more often	15	
3.4b What is the overall hand hygiene compliance rate according to the WHO Hand Hygiene Observation tool (or similar technique) in your facility? **Choose one answer**	≤ 30%	0	→ Guide to Implementation II.3<br>→ Observation form<br>→ Data Entry Analysis tools<br>→ Instructions for Data Entry and Analysis<br>→ Epi Info™ software[9]<br>→ Data Summary Report Framework
	31 – 40%	5	
	41 – 50%	10	
	51 – 60%	15	
	61 – 70%	20	
	71 – 80%	25	
	≥ 81%	30	
**3.5 Feedback**			
3.5a **Immediate feedback** Is immediate feedback given to health-care workers at the end of each hand hygiene compliance observation session?	No	0	→ Guide to Implementation II.3<br>→ Observation and Basic Compliance Calculation forms
	Yes	5	
3.5b **Systematic feedback** Is regular (at least 6 monthly) feedback of data related to hand hygiene indicators with demonstration of trends over time given to:			→ Data Summary Report Framework<br>→ Guide to Implementation II.3
3.5b.i Health-care workers?	No	0	
	Yes	7.5	
3.5b.ii Facility leadership?	No	0	
	Yes	7.5	
	**Evaluation and Feedback subtotal**	**/100**	

9. Epi InfoTM: This software can be downloaded free of charge from the CDC website (http://www.cdc.gov/epiinfo/)

All reasonable precautions have been taken by the World Health Organization to verify the information contained in this document. However, the published material is being distributed without warranty of any kind, either expressed or implied. The responsibility for the interpretation and use of the material lies with the reader. In no event shall the World Health Organization be liable for damages arising from its use.
WHO acknowledges the Hôpitaux Universitaires de Genève (HUG), in particular the members of the Infection Control Programme, for their active participation in developing this material.

4. Reminders in the Workplace

Question	Answer	Score	WHO improvement tools
**4.1** Are the following posters (or locally produced equivalent with similar content) displayed?			→ Guide to Implementation II.4
4.1a Poster explaining the indications for hand hygiene **Choose one answer**	Not displayed	0	→ Your 5 Moments for Hand Hygiene (Poster)
	Displayed in some wards/treatment areas	15	
	Displayed in most wards/treatment areas	20	
	Displayed in all wards/treatment areas	25	
4.1b Poster explaining the correct use of handrub **Choose one answer**	Not displayed	0	→ How to Handrub (Poster)
	Displayed in some wards/treatment areas	5	
	Displayed in most wards/treatment areas	10	
	Displayed in all wards/treatment areas	15	
4.1c Poster explaining correct hand-washing technique **Choose one answer**	Not displayed	0	→ How to Handwash (Poster)
	Displayed in some wards/treatment areas	5	
	Displayed in most wards/treatment areas	7.5	
	Displayed at every sink in all wards/treatment areas	10	
**4.2** How frequently does a systematic audit of all posters for evidence of damage occur, with replacement as required? **Choose one answer**	Never	0	→ Guide to Implementation II.4
	At least annually	10	
	Every 2-3 months	15	
**4.3** Is hand hygiene promotion undertaken by displaying and regularly updating posters other than those mentioned above?	No	0	→ Guide to Implementation II.4
	Yes	10	
**4.4** Are hand hygiene information leaflets available on wards?	No	0	→ Hand Hygiene: When and How Leaflet
	Yes	10	→ Guide to Implementation II.4
**4.5** Are other workplace reminders located throughout the facility? (e.g. hand hygiene campaign screensavers, badges, stickers, etc)	No	0	→ SAVE LIVES: Clean Your Hands Screensaver → Guide to Implementation II.4
	Yes	15	
	**Reminders in the Workplace subtotal**	**/100**	

All reasonable precautions have been taken by the World Health Organization to verify the information contained in this document. However, the published material is being distributed without warranty of any kind, either expressed or implied. The responsibility for the interpretation and use of the material lies with the reader. In no event shall the World Health Organization be liable for damages arising from its use.
WHO acknowledges the Hôpitaux Universitaires de Genève (HUG), in particular the members of the Infection Control Programme, for their active participation in developing this material.

5. Institutional Safety Climate for Hand Hygiene

Question	Answer	Score	WHO improvement tools
**5.1** With regard to a hand hygiene team[10] that is dedicated to the promotion and implementation of optimal hand hygiene practice in your facility:			→ Guide to Implementation II.5
5.1a Is such a team established?	No	0	
	Yes	5	
5.1b Does this team meet on a regular basis (at least monthly)?	No	0	
	Yes	5	
5.1c Does this team have dedicated time to conduct active hand hygiene promotion? (e.g. teaching monitoring hand hygiene performance, organizing new activities)	No	0	
	Yes	5	
**5.2** Have the following members of the facility leadership made a clear commitment to support hand hygiene improvement? (e.g. a written or verbal commitment to hand hygiene promotion received by the majority of health-care workers)			→ Template Letter to Advocate Hand Hygiene to Managers<br>→ Template Letter to communicate Hand Hygiene Initiatives to Managers<br>→ Guide to Implementation II.5
5.2a Chief executive officer	No	0	
	Yes	10	
5.2b Medical director	No	0	
	Yes	5	
5.2c Director of nursing	No	0	
	Yes	5	
**5.3** Has a clear plan for the promotion of hand hygiene throughout the entire facility for the 5 May (Save Lives Clean Your Hands Annual Initiative) been established ?	No	0	→ Sustaining Improvement – Additional Activities for Consideration by Health-Care Facilities<br>→ Guide to Implementation II.5
	Yes	10	
**5.4** Are systems for identification of Hand Hygiene Leaders from all disciplines in place?			
5.4a A system for designation of Hand Hygiene champions[11]	No	0	
	Yes	5	
5.4b A system for recognition and utilisation of Hand Hygiene role models[12]	No	0	
	Yes	5	
**5.5** Regarding patient involvement in hand hygiene promotion:			→ Guidance on Engaging Patients and Patient Organizations in Hand Hygiene Initiatives<br>→ Guide to Implementation II.5
5.5a Are patients informed about the importance of hand hygiene? (e.g. with a leaflet)	No	0	
	Yes	5	
5.5b Has a formalised programme of patient engagement been undertaken?	No	0	
	Yes	10	
**5.6** Are initiatives to support local continuous improvement being applied in your facility, for example:			→ Sustaining Improvement – Additional Activities for Consideration by Health-Care Facilities<br>→ Guide to Implementation II.5
5.6a Hand hygiene E-learning tools	No	0	
	Yes	5	
5.6b A hand hygiene institutional target to be achieved is established each year	No	0	
	Yes	5	
5.6c A system for intra-institutional sharing of reliable and tested local innovations	No	0	
	Yes	5	
5.6d Communications that regularly mention hand hygiene e.g. facility newsletter, clinical meetings	No	0	
	Yes	5	
5.6e System for personal accountability[13]	No	0	
	Yes	5	
5.6f A Buddy system[14] for new employees	No	0	
	Yes	5	
**Institutional Safety Climate subtotal**		/100	

All reasonable precautions have been taken by the World Health Organization to verify the information contained in this document. However, the published material is being distributed without warranty of any kind, either expressed or implied. The responsibility for the interpretation and use of the material lies with the reader. In no event shall the World Health Organization be liable for damages arising from its use.
WHO acknowledges the Hôpitaux Universitaires de Genève (HUG), in particular the members of the Infection Control Programme, for their active participation in developing this material.

10. Hand hygiene team: The make-up of this team will vary. It is likely to most frequently consist of an infection control unit, but may range (depending on resources available) from a single person with the role of managing the hand hygiene programme, to a group of staff members from various departments within the facility with meetings dedicated to the hand hygiene programme.

11. Hand hygiene champion: A person who is an advocate for the causes of patient safety and hand hygiene standards and takes on responsibility for publicizing a project in his/her ward and/or facility-wide.

12. Hand hygiene role model: A person who serves as an example, whose behaviour is emulated by others. In particular, a hand hygiene role model should have a hand hygiene compliance rate of at least 80%, be able to remind others to comply, and be able to teach practically about the WHO 5 Moments for Hand Hygiene concept.

13. System for personal accountability: explicit actions are in place to stimulate health-care workers to be accountable for their behaviour with regard to hand hygiene practices. Examples are notification by observers or infection control professionals, reproaches by peers, and reports to higher level facility authorities, with possible consequences on the individual evaluation.

14. Buddy system: A programme in which each new health-care worker is coupled with an established, trained health-care worker who takes responsibility for introducing them to the hand hygiene culture of the health-care setting (including practical training on indications and technique for performing hand hygiene, and explanation of hand hygiene promotion initiatives within the facility).

All reasonable precautions have been taken by the World Health Organization to verify the information contained in this document. However, the published material is being distributed without warranty of any kind, either expressed or implied. The responsibility for the interpretation and use of the material lies with the reader. In no event shall the World Health Organization be liable for damages arising from its use.
WHO acknowledges the Hôpitaux Universitaires de Genève (HUG), in particular the members of the Infection Control Programme, for their active participation in developing this material.

Interpretation: A Four Step Process

1.
Add up your points.

Score	
**Component**	**Subtotal**
1. System Change	
2. Education and Training	
3. Evaluation and Feedback	
4. Reminders in the Workplace	
5. Institutional Safety Climate	
**Total**	

2.
Determine the assigned 'Hand Hygiene Level' for your facility.

**Total Score** (range)	**Hand Hygiene Level**
0 - 125	Inadequate
126 - 250	Basic
251 - 375	Intermediate (or Consolidation)
376 - 500	Advanced (or Embedding)

3.
If your facility has reached the Advanced level, then complete the Leadership section overleaf.

(otherwise go to Step 4).

4.
Review the areas identified by this evaluation as requiring improvement in your facility and develop an action plan to address them (starting with the relevant WHO improvement tools listed). Keep a copy of this assessment to compare with repeated uses in the future.

All reasonable precautions have been taken by the World Health Organization to verify the information contained in this document. However, the published material is being distributed without warranty of any kind, either expressed or implied. The responsibility for the interpretation and use of the material lies with the reader. In no event shall the World Health Organization be liable for damages arising from its use.
WHO acknowledges the Hôpitaux Universitaires de Genève (HUG), in particular the members of the Infection Control Programme, for their active participation in developing this material.

Hand Hygiene Self-Assessment Framework 2010

Leadership Criteria	Answer (circle one)	
**System Change**		
Has a cost-benefit analysis of infrastructure changes required for the performance of optimal hand hygiene at the point of care been performed?	Yes	No
Does alcohol-based handrubbing account for at least 80% of hand hygiene actions performed in your facility?	Yes	No
**Training and Education**		
Has the hand hygiene team undertaken training of representatives from other facilities in the area of hand hygiene promotion?	Yes	No
Have hand hygiene principles been incorporated into local medical and nursing educational curricula?	Yes	No
**Evaluation and Feedback**		
Are specific healthcare associated infections (HCAIs) monitored? (eg. *Staphylococcus aureus* bacteremia, Gram negative bacteremia, device-related infections)	Yes	No
Is a system in place for monitoring of HCAI in high risk-settings? (e.g. intensive care and neonatal units)	Yes	No
Is a facility-wide prevalence survey of HCAI performed (at least) annually?	Yes	No
Are HCAI rates presented to facility leadership and to health-care workers in conjunction with hand hygiene compliance rates?	Yes	No
Is structured evaluation undertaken to understand the obstacles to optimal hand hygiene compliance and the causes of HCAI at the local level, and results reported to the facility leadership?	Yes	No
**Reminders in the Workplace**		
Is a system in place for creation of new posters designed by local health-care workers?	Yes	No
Are posters created in your facility used in other facilities?	Yes	No
Have innovative types of hand hygiene reminders been developed and tested at the facility?	Yes	No
**Institutional Safety Climate**		
Has a local hand hygiene research agenda addressing issues identified by the WHO Guidelines as requiring further investigation been developed?	Yes	No
Has your facility participated actively in publications or conference presentations (oral or poster) in the area of hand hygiene?	Yes	No
Are patients invited to remind health-care workers to perform hand hygiene?	Yes	No
Are patients and visitors educated to correctly perform hand hygiene?	Yes	No
Does your facility contribute to and support the national hand hygiene campaign (if existing)?	Yes	No
Is impact evaluation of the hand hygiene campaign incorporated into forward planning of the infection control programme?	Yes	No
Does your facility set an annual target for improvement of hand hygiene compliance facility-wide?	Yes	No
If the facility has such a target, was it achieved last year?	Yes	No
**Total**	/20	

Your facility has reached the Hand Hygiene Leadership level if you answered "yes" to at least one leadership criteria per category and its total leadership score is 12 or more. Congratulations and thank you!

All reasonable precautions have been taken by the World Health Organization to verify the information contained in this document. However, the published material is being distributed without warranty of any kind, either expressed or implied. The responsibility for the interpretation and use of the material lies with the reader. In no event shall the World Health Organization be liable for damages arising from its use.
WHO acknowledges the Hôpitaux Universitaires de Genève (HUG), in particular the members of the Infection Control Programme, for their active participation in developing this material.

Supplementary File S2: Survey selection and weighting procedure

Survey selection

In figure 1 in the main manuscript, all survey selection steps are displayed, detailed explanations can be found below.

Selection step 1

As a first step, all survey responses received through the publicly available online survey link, or the dedicated survey link for nationally coordinated data collection were screened for completeness. Since the survey link could be used multiple times by the same respondent, certain respondents submitted “test” forms, to go through all the questions. Other respondents started multiple surveys, or only answered a very limited number of questions. To exclude these erroneously submitted surveys in a standardized way, only surveys that completed at least one element (System Change, Training and Education, Evaluation and Feedback, Reminders in the Workplace, and Institutional Safety Climate) of the Hand Hygiene Self-Assessment Framework were selected and data included in the analysis of the specific elements.

Selection step 2

Since participation in the Hand Hygiene Self-Assessment Framework global survey was on an individual basis, single healthcare facilities (HCFs) could have multiple survey responses. To eliminate duplication, single HCF responses were identified through a geospatial clustering algorithm, and selected through a pre-defined strategy. Geospatial clustering was based on hospital name (free text), city (free text) and country (drop-down menu), using R packages::functions ‘ggmap::geocode’, ‘SP::SpatialPointsDataFrame’, ‘geosphere::distm’, ‘stats::hclust’, and ‘stats::cutree’ with a cut-off of 100 meters If geospatial clusters were identified and it was manually verified that responses came from the same HCF, the following strategy was applied:

For nationally coordinated data collection, WHO National Offices were requested to select the most appropriate response (based on direct contact with HCF/name of the respondent/email address of respondent). Otherwise the survey response was selected based on the sequential application of the following criteria:

1. Most indicators (questions) completed
2. Most elements (System Change, Training and Education, Evaluation and Feedback, Reminders in the Workplace, and Institutional Safety Climate) fully completed
3. Part of nationally coordinated data collection (response through dedicated survey link)
4. Random selection

Improving global representativeness through selection step 3 and weighting

As it was a self-selecting survey, the number of responses per country varied widely. Therefore, two strategies were applied to improve global representativeness; responses from countries with a ratio of number of survey responses per capita in the lowest ventile were excluded from the analysis, and post-stratification weighting was applied.

Since it is likely that the HCFs dedicated to hand hygiene implementation would be most motivated to respond within a country, this could introduce an important bias and reduce global representativeness. Therefore, it was decided to exclude responses from countries with very low response rates. This was expressed per capita to take into account population size, as smaller countries will have less HCFs and thus a lower chance of high numbers of responses. This selection step is even more important, due to the weighting procedure, explained below. This methodology assigned an equal importance to each country, and therefore a large weight would be given to individual responses, if a country had very few responses.

The weighting procedure considered all important strata: country, World Bank country income level (World Bank GNI/capita 2019; low<=$1,035, lower middle $1,036 - $4,045, upper middle $4,046-12,535, high >$12,535) (https://datahelpdesk.worldbank.org/knowledgebase/topics/19280-country-classification), WHO region (Africa, Eastern Mediterranean, Europe, South East Asia, Western Pacific), facility level (primary/ secondary/ tertiary care hospitals), and type of facility (private/ public hospitals). To determine weights, R's anesrake package was applied, with the lowest weight cap possible for convergence (cap=40); each country was assigned an equal importance (1/total number of participating countries), for region and income level the proportion of countries in each category determined overall importance. The marginal distribution for level of facility was based on data from 134 countries reported to the WHO Global Health Observatory (https://www.who.int/data/gho/data/themes/topics/ indicator-groups/indicator-group-details/GHO/health-infrastructure) in 2013 (primary 72%, secondary 13%, tertiary 10%, other 5%), while for type of facility equal weights were assigned to responses from public and private facilities (private 48%, public 48%, other 4%), as no reliable data on distribution was available and this would guarantee equal weight.

Supplementary File S3: Generalized Estimating Equations (GEE) showing the independent association between element-specific scores for the Hand Hygiene Self-Assessment Framework (HHSAF) survey 2019 and country and healthcare facility characteristics.

Country was included as cluster, post-stratification weights (for country, country income level, region, facility type and level) were applied, and only surveys that completed the respective Element were included.

Supplementary Table S3.1: GEE for System Change (N=3165)

Variable	Coefficient (95% CI)	P-value
**World Bank Income level** (reference=High)		
Upper Middle	-9.9 (-19.5 – -0.3)	**0.044**
Lower Middle	-22.2 (-33.0 – -11.4)	**<0.0001**
Low	-35.2 (-48.4 – -22.5)	**<0.0001**
**WHO Region** (reference= Africa)		
Eastern Mediterranean	-2.0 (-12.1 – 8.1)	0.69
Europe	-1.2 (-14.1 – 11.7)	0.85
Americas	-4.9 (-15.7 – 5.9)	0.37
South East Asia	-3.6 (-14.2 – 7.0)	0.51
Western Pacific	-2.5 (-14.6 – 9.5)	0.68
**Nationally coordinated data collection** (reference= No)		
Yes	-6.2 (-16.2 – 3.9)	0.23
**Facility level** (reference=Primary)		
Secondary	-6.0 (-14.3 – 2.2)	0.15
Tertiary	2.8 (-6.0 – 11.6)	0.53
Other	1.4 (-5.4 – 8.2)	0.68
**Type of facility** (reference=public)	*Reference*	
Private	19.1 (12.5 – 25.7)	**<0.0001**
Other	13.4 (5.1 – 21.6)	**0.0016**

Supplementary Table S3.2: GEE for Training and Education (N=3161)

Variable	Coefficient (95% CI)	P-value
**World Bank Income level** (reference=High)		
Upper Middle	-7.4 (-17.8 – 2.9)	0.16
Lower Middle	-13.4 (-26.3 – -0.5)	**0.041**
Low	-32.1 (-47.2 – -16.9)	**<0.0001**
**WHO Region** (reference= Africa)		
Eastern Mediterranean	7.0 (-6.3 – 20.2)	0.30
Europe	-2.4 (-18.2 – 13.4)	0.76
Americas	-2.1 (-17.0 – 12.8)	0.79
South East Asia	-10.2 (-27.9 – 7.4)	0.26
Western Pacific	2.5 (-16.2 – 21.2)	0.79
**Nationally coordinated data collection** (reference= No)		
Yes	-0.71 (-8.8 – 7.4)	0.86
**Facility level** (reference=Primary)		
Secondary	-6.1 (-17.3 – 5.0)	0.28
Tertiary	-0.4 (-12.0 – 11.3)	0.95
Other	3.7 (-3.3 – 10.6)	0.30
**Type of facility** (reference=public)		
Private	14.7 (6.1 – 23.4)	**0.0008**
Other	6.8 (-3.1 – 16.6)	0.18

Supplementary Table S3.3: GEE for Evaluation and Feedback (N=3137)

Variable	Coefficient (95% CI)	P-value
**World Bank Income level** (reference=High)		
Upper Middle	-2.0 (-14.3 – 10.3)	0.75
Lower Middle	-14.3 (-30.9 – 2.3)	0.092
Low	-22.3 (-40.6 – -4.0)	**0.015**
**WHO Region** (reference= Africa)		
Eastern Mediterranean	5.1 (-10.9 – 21.1)	0.53
Europe	-3.3 (-21.8 – 15.3)	0.73
Americas	5.1 (-13.0 – 23.2)	0.58
South East Asia	-1.3 (-21.1 – 18.5)	0.90
Western Pacific	10.4 (-7.9 – 28.6)	0.27
**Nationally coordinated data collection** (reference= No)		
Yes	-6.8 (-16.5 – 2.8)	0.17
**Facility level** (reference=Primary)		
Secondary	-10.5 (-25.2 – 4.1)	0.16
Tertiary	-3.4 (-16.9 – 10.1)	0.62
Other	-0.5 (-8.4 – 7.4)	0.90
**Type of facility** (reference=public)		
Private	15.4 (5.5 – 25.3)	**0.0022**
Other	18.1 (2.5 – 33.6)	**0.022**

Supplementary Table S3.4: GEE for Reminders in the Workplace (N=3153)

Variable	Coefficient (95% CI)	P-value
**World Bank Income level** (reference=High)		
Upper Middle	1.9 (-8.3 – 12.0)	0.72
Lower Middle	-11.3 (-25.0 – 2.3)	0.10
Low	-26.1 (-42.9 – -9.4)	**0.0022**
**WHO Region** (reference= Africa)		
Eastern Mediterranean	8.1 (-4.3 – 20.6)	0.20
Europe	-7.2 (-22.6 – 8.1)	0.35
Americas	-7.3 (-22.5 – 8.0)	0.35
South East Asia	-11.9 (-32.7 – 9.0)	0.26
Western Pacific	2.4 (-12.2 – 16.9)	0.75
**Nationally coordinated data collection** (reference= No)		
Yes	-0.2 (-7.8 – 7.3)	0.95
**Facility level** (reference=Primary)		
Secondary	-4.1 (-16.1 – 8.0)	0.51
Tertiary	0.6 (-11.8 – 13.0)	0.92
Other	4.0 (-3.8 – 11.8)	0.32
**Type of facility** (reference=public)		
Private	12.5 (3.9 – 21.2)	**0.0046**
Other	11.5 (1.6 – 21.4)	**0.023**

Supplementary Table S3.5: GEE for Institutional Safety Climate (N=3102)

Variable	Coefficient (95% CI)	P-value
**World Bank Income level** (reference=High)		
Upper Middle	-2.7 (-14.5 – 9.0)	0.65
Lower Middle	-13.9 (-26.7 – -1.2)	**0.032**
Low	-21.9 (-33.9 – -9.9)	**0.0004**
**WHO Region** (reference= Africa)		
Eastern Mediterranean	-1.7 (-13.7 – 10.3)	0.78
Europe	-12.3 (-26.8 – 2.2)	0.096
Americas	-5.0 (-19.2 – 9.2)	0.49
South East Asia	-15.2 (-35.1 – 4.8)	0.14
Western Pacific	-3.0 (-16.5 – 10.5)	0.67
**Nationally coordinated data collection** (reference= No)		
Yes	-7.8 (-14.0 – -1.6)	**0.013**
**Facility level** (reference=Primary)		
Secondary	-4.9 (-15.1– 5.3)	0.34
Tertiary	1.6 (-8.2 – 11.3)	0.76
Other	5.1 (-2.0 – 12.3)	0.16
**Type of facility** (reference=public)		
Private	14.2 (6.5 – 22.0)	**0.0003**
Other	11.5 (2.0 – 20.9)	**0.017**

Supplementary File S4: Indicator responses for the Hand Hygiene Self-Assessment Framework (HHSAF) survey 2019 specified per element, weighted for country, country income level, region, facility type and level.

Only includes survey responses without missing values for the respective element.

Table S4.1: System Change: Weighted HHSAF responses specified per question
*Only includes surveys without missing values for element 1

	Nw		ABHR availability		Sink:bed ratio		Water supply	Soap at sink	Single use towels at sink	Dedicated ABHR budget	Infrastructure improvement plan*
**Overall**	**3189**	**Response**	**Nw (%)**	**Response**	**Nw (%)**	**Response**	**Nw (%)**	**Nw (%)**	**Nw (%)**	**Nw (%)**	**Nw (%)**
		NA	117 (3.7)	<1:10	410 (12.9)	No	123 (3.9)	331 (10.4)	1184 (37.1)	590 (18.5)	852 (26.7)
		Available	119 (3.7)	1:10	897 (28.1)	Yes	3066 (96.1)	2858 (89.6)	2005 (62.9)	2600 (81.5)	1323 (41.5)
		+ proven	415 (13.0)	1:10 & 1:1	1882 (59.0)						
		+ cont supply	240 (7.5)								
		+ PoC	676 (21.2)								
		+ cont supply PoC	1623 (50.9)								
**WHO region**											
Africa	638	NA	52 (8.2)	<1:10	174 (27.3)	No	75 (11.7)	49 (7.7)	443 (69.5)	157 (24.6)	229 (35.9)
		Available	26 (4.1)	1:10	153 (24.0)	Yes	564 (88.3)	589 (92.3)	195 (30.5)	481 (75.4)	312 (48.8)
		+ proven	152 (23.7)	1:10 & 1:1	311 (48.7)						
		+ cont supply	36 (5.6)								
		+ PoC	130 (20.4)								
		+ cont supply PoC	242 (38.0)								

	Nw		ABHR availability		Sink:bed ratio		Water supply	Soap at sink	Single use towels at sink	Dedicated ABHR budget	Infrastructure improvement plan*
Eastern Mediterranean	579	NA	7 (1.2)	<1:10	91 (15.8)	No	14 (2.3)	72 (12.5)	193 (33.3)	72 (12.5)	149 (25.7)
		Available	12 (2.1)	1:10	136 (23.5)	Yes	565 (97.7)	506 (87.5)	386 (66.7)	507 (87.5)	214 (36.9)
		+ proven	41 (7.2)	1:10 & 1:1	351 (60.7)						
		+ cont supply	54 (9.3)								
		+ PoC	165 (28.5)								
		+ cont supply PoC	300 (51.8)								
Europe	634	NA	1 (0.2)	<1:10	12 (1.9)	No	0 (0)	19 (3.1)	99 (15.6)	80 (12.5)	100 (15.8)
		Available	29 (4.5)	1:10	147 (23.2)	Yes	634 (100.0)	615 (96.9)	535 (84.4)	555 (87.5)	213 (33.7)
		+ proven	25 (3.9)	1:10 & 1:1	475 (74.9)						
		+ cont supply	60 (9.5)								
		+ PoC	67 (10.5)								
		+ cont supply PoC	452 (71.3)								
Americas	641	NA	54 (8.5)	<1:10	38 (5.9)	No	14 (2.2)	124 (19.3)	243 (37.9)	171 (26.6)	226 (35.3)
		Available	30 (4.6)	1:10	275 (42.8)	Yes	627 (97.8)	517 (80.7)	398 (62.1)	470 (73.4)	245 (38.2)
		+ proven	74 (11.6)	1:10 & 1:1	328 (51.2)						
		+ cont supply	24 (3.8)								
		+ PoC	173 (27.0)								
		+ cont supply PoC	286 (44.6)								
South East Asia	233	NA		<1:10	57 (24.6)	No	18 (7.9)	22 (9.4)	106 (45.7)	43 (18.4)	74 (31.8)

	Nw		ABHR availability		Sink:bed ratio		Water supply	Soap at sink	Single use towels at sink	Dedicated ABHR budget	Infrastructure improvement plan*
		Available	21 (8.9)	1:10	107 (45.9)	Yes	215 (92.1)	211 (90.6)	127 (54.3)	190 (81.6)	138 (59.1)
		+ proven	47 (20.0)	1:10 & 1:1	69 (29.4)						
		+ cont supply	12 (5.1)								
		+ PoC	77 (33.2)								
		+ cont supply PoC	77 (32.9)								
Western Pacific	464	NA	2 (0.5)	<1:10	37 (8.0)	No	2 (0.5)	45 (9.7)	100 (21.5)	68 (14.6)	73 (15.7)
		Available	1 (0.2)	1:10	78 (16.9)	Yes	462 (99.5)	419 (90.3)	364 (78.5)	396 (85.4)	201 (43.4)
		+ proven	76 (16.4)	1:10 & 1:1	348 (75.1)						
		+ cont supply	54 (11.5)								
		+ PoC	64 (13.9)								
		+ cont supply PoC	266 (57.4)								
**Income Level**											
Low-income	347	NA	24 (6.8)	<1:10	166 (47.8)	No	75 (21.6)	58 (16.8)	318 (91.8)	117 (33.8)	134 (38.7)
		Available	34 (9.9)	1:10	63 (18.3)	Yes	272 (78.4)	289 (83.2)	29 (8.2)	230 (66.2)	212 (61.0)
		+ proven	92 (26.4)	1:10 & 1:1	118 (33.9)						
		+ cont supply	27 (7.8)								
		+ PoC	112 (32.4)								
		+ cont supply PoC	58 (16.7)								
Lower Middle-income	697	NA	38 (5.4)	<1:10	128 (18.3)	No	32 (4.6)	95 (13.6)	451 (64.6)	218 (31.3)	303 (43.5)

	Nw		ABHR availability		Sink:bed ratio		Water supply	Soap at sink	Single use towels at sink	Dedicated ABHR budget	Infrastructure improvement plan*
		Available	18 (2.7)	1:10	291 (41.8)	Yes	665 (95.4)	602 (86.4)	246 (35.4)	479 (68.7)	322 (46.2)
		+ proven	169 (24.3)	1:10 & 1:1	278 (39.9)						
		+ cont supply	74 (10.7)								
		+ PoC	154 (22.1)								
		+ cont supply PoC	243 (34.9)								
Upper Middle-income	1038	NA	38 (3.7)	<1:10	87 (8.3)	No	14 (1.3)	82 (7.9)	279 (26.8)	150 (14.5)	278 (26.8)
		Available	42 (4.0)	1:10	304 (29.2)	Yes	1024 (98.7)	956 (92.1)	759 (73.2)	888 (85.5)	441 (42.5)
		+ proven	107 (10.3)	1:10 & 1:1	648 (62.4)						
		+ cont supply	96 (9.2)								
		+ PoC	261 (25.1)								
		+ cont supply PoC	494 (47.6)								
High-income	1107	NA	18 (1.6)	<1:10	30 (2.7)	No	2 (0.2)	96 (8.7)	136 (12.3)	104 (9.4)	136 (12.3)
		Available	24 (2.1)	1:10	239 (21.5)	Yes	1105 (99.8)	1011 (91.3)	971 (87.7)	1003 (90.6)	348 (31.5)
		+ proven	47 (4.2)	1:10 & 1:1	839 (75.8)						
		+ cont supply	43 (3.9)								
		+ PoC	149 (13.4)								
		+ cont supply PoC	828 (74.7)								
**Nationally coordinated**											
Yes	433	NA	28 (6.4)	<1:10	106 (24.5)	No	39 (9.1)	77 (17.7)	217 (47.2)	101 (23.3)	110 (25.3)

	Nw		ABHR availability		Sink:bed ratio		Water supply	Soap at sink	Single use towels at sink	Dedicated ABHR budget	Infrastructure improvement plan*
		Available	49 (11.3)	1:10	146 (33.8)	Yes	393 (90.9)	356 (82.3)	228 (52.8)	332 (76.7)	228 (52.8)
		+ proven	67 (15.6)	1:10 & 1:1	180 (41.7)						
		+ cont supply	19 (4.5)								
		+ PoC	89 (20.5)								
		+ cont supply PoC	180 (41.7)								
No	2757	NA	90 (3.2)	<1:10	304 (11.0)	No	84 (3.0)	255 (9.2)	980 (35.5)	489 (17.7)	742 (26.9)
		Available	70 (2.5)	1:10	751 (27.2)	Yes	2673 (97.0)	2502 (90.8)	1777 (64.5)	2268 (82.3)	1095 (39.7)
		+ proven	347 (12.6)	1:10 & 1:1	1702 (61.7)						
		+ cont supply	220 (8.0)								
		+ PoC	587 (21.3)								
		+ cont supply PoC	1443 (52.3)								
**Facility Type**											
Private	1524	NA	4 (0.2)	<1:10	24 (1.6)	No	2 (0.2)	39 (2.5)	348 (22.8)	149 (9.8)	199 (13.0)
		Available	2 (0.1)	1:10	264 (17.3)	Yes	1522 (99.8)	1486 (97.5)	1177 (77.2)	1375 (90.2)	589 (38.6)
		+ proven	105 (6.9)	1:10 & 1:1	1237 (81.1)						
		+ cont supply	94 (6.2)								
		+ PoC	287 (18.8)								
		+ cont supply PoC	1034 (67.8)								
Public	1537	NA	105 (6.8)	<1:10	350 (22.8)	No	117 (7.6)	289 (18.8)	800 (52.1)	400 (26.0)	622 (40.5)

	Nw		ABHR availability		Sink:bed ratio		Water supply	Soap at sink	Single use towels at sink	Dedicated ABHR budget	Infrastructure improvement plan*
		Available	116 (7.6)	1:10	604 (39.3)	Yes	1420 (92.4)	1248 (81.2)	737 (47.9)	1137 (74.0)	686 (44.7)
		+ proven	296 (19.3)	1:10 & 1:1	582 (37.9)						
		+ cont supply	142 (9.2)								
		+ PoC	353 (22.9)								
		+ cont supply PoC	525 (34.2)								
Other	128	NA	9 (7.0)	<1:10	35 (27.6)	No	4 (3.1)	4 (2.9)	36 (28.1)	41 (31.7)	31 (24.3)
		Available	0 (0.2)	1:10	30 (23.1)	Yes	124 (96.9)	124 (97.1)	92 (71.9)	88 (68.3)	48 (37.3)
		+ proven	14 (10.8)	1:10 & 1:1	63 (49.3)						
		+ cont supply	4 (3.4)								
		+ PoC	37 (28.7)								
		+ cont supply PoC	64 (49.9)								
**Facility Level**											
Primary	2295	NA	100 (4.3)	<1:10	263 (11.5)	No	98 (4.3)	179 (7.8)	816 (35.6)	420 (18.3)	606 (26.4)
		Available	95 (4.1)	1:10	667 (29.0)	Yes	2197 (95.7)	2116 (92.2)	1479 (64.4)	1876 (81.7)	971 (42.3)
		+ proven	300 (13.1)	1:10 & 1:1	1366 (59.5)						
		+ cont supply	111 (4.9)								
		+ PoC	515 (22.4)								
		+ cont supply PoC	1174 (51.1)								
Secondary	414	NA	9 (2.2)	<1:10	51 (12.3)	No	18 (4.4)	91 (21.9)	187 (45.3)	119 (28.7)	113 (27.3)

	Nw		ABHR availability		Sink:bed ratio		Water supply	Soap at sink	Single use towels at sink	Dedicated ABHR budget	Infrastructure improvement plan*
		Available	17 (4.2)	1:10	101 (24.4)	Yes	395 (95.6)	323 (78.1)	226 (54.7)	295 (71.3)	192 (46.4)
		+ proven	85 (20.7)	1:10 & 1:1	262 (63.3)						
		+ cont supply	49 (11.9)								
		+ PoC	77 (18.6)								
		+ cont supply PoC	176 (42.5)								
Tertiary	320	NA	4 (1.3)	<1:10	74 (23.0)	No	5 (1.5)	48 (14.9)	136 (42.3)	39 (12.3)	110 (34.3)
		Available	3 (1.0)	1:10	99 (31.0)	Yes	316 (98.5)	273 (85.1)	185 (57.7)	281 (87.7)	111 (34.7)
		+ proven	16 (5.1)	1:10 & 1:1	148 (46.0)						
		+ cont supply	65 (20.2)								
		+ PoC	71 (22.1)								
		+ cont supply PoC	161 (50.3)								
Other	160	NA	5 (2.8)	<1:10	23 (14.4)	No	2 (1.0)	14 (9.0)	45 (28.0)	12 (7.4)	23 (14.5)
		Available	3 (1.8)	1:10	30 (18.6)	Yes	159 (99.0)	146 (91.0)	115 (72.0)	148 (92.6)	49 (30.8)
		+ proven	13 (8.1)	1:10 & 1:1	107 (67.0)						
		+ cont supply	14 (8.8)								
		+ PoC	14 (8.5)								
		+ cont supply PoC	112 (70.0)								

Nw, weighted frequency; ABHR, alcohol-based handrub; IPC, infection prevention and control; PoC, point of care
* This question was completed by 3189 healthcare facilities with a score<100 for the first 6 questions

Table S4.2: Element 2 ‘Training and Education’: Weighted HHSAF responses specified per question
*Only includes surveys without missing values for element 2

	Nw		HH training HCWs		Training registry	WHO HH summary available	WHO Technical manual available	WHO HH brochure available	WHO glove use leaflet available	Active HH trainer	Training of HH observers	Budget for training
**Overall**	**3182**	**Response**	**Nw (%)**	**Response**	**Nw (%)**	**Nw (%)**	**Nw (%)**	**Nw (%)**	**Nw (%)**	**Nw (%)**	**Nw (%)**	**Nw (%)**
		Never	91 (2.9)	No	1038 (32.6)	827 (26.0)	1012 (31.8)	868 (27.3)	1230 (38.6)	421 (13.2)	1227 (38.6)	1819 (57.2)
		Once	526 (16.5)	Yes	2144 (67.4)	2355 (74.0)	2170 (68.2)	2314 (72.7)	1952 (61.4)	2761 (86.8)	1954 (61.4)	1362 (42.8)
		Regular	1034 (32.5)									
		Mandatory	1531 (48.1)									
**WHO region**												
Africa	640	Never	30 (4.7)	No	336 (52.5)	252 (39.3)	339 (52.9)	263 (41.1)	373 (58.3)	80 (12.4)	352 (54.9)	370 (57.8)
		Once	153 (23.9)	Yes	304 (47.5)	388 (60.7)	301 (47.1)	377 (58.9)	267 (41.7)	561 (87.6)	289 (45.1)	270 (42.2)
		Regular	217 (33.8)									
		Mandatory	240 (37.5)									
Eastern Mediterranean	577	Never	6 (1.1)	No	108 (18.6)	54 (9.3)	131 (22.6)	81 (14.1)	156 (27.1)	56 (9.7)	182 (31.4)	301 (52.1)
		Once	87 (15.0)	Yes	470 (81.4)	523 (90.7)	447 (77.4)	496 (85.9)	421 (72.9)	521 (90.3)	396 (68.6)	277 (47.9)
		Regular	162 (28.1)									
		Mandatory	322 (55.8)									
Europe	640	Never	0 (0)	No	181 (28.2)	91 (14.3)	107 (16.6)	106 (16.6)	154 (24.1)	68 (10.6)	206 (32.1)	360 (56.3)
		Once	114 (17.8)	Yes	459 (71.8)	549 (85.7)	533 (83.4)	534 (83.4)	486 (75.9)	572 (89.4)	434 (67.9)	280 (43.7)
		Regular	206 (32.2)									
		Mandatory	320 (50.0)									

	Nw		HH training HCWs		Training registry	WHO HH summary available	WHO Technical manual available	WHO HH brochure available	WHO glove use leaflet available	Active HH trainer	Training of HH observers	Budget for training
Americas	635	Never	14 (2.3)	No	223 (35.0)	222 (34.9)	208 (32.8)	169 (26.6)	219 (34.5)	80 (12.7)	272 (42.8)	456 (71.7)
		Once	55 (8.6)	Yes	413 (65.0)	413 (65.1)	427 (67.2)	466 (73.4)	416 (65.5)	555 (87.3)	363 (57.2)	180 (28.3)
		Regular	254 (40)									
		Mandatory	312 (49.1)									
South East Asia	227	Never	39 (17.4)	No	92 (40.5)	44 (19.3)	69 (30.3)	104 (45.7)	132 (58.1)	92 (40.4)	100 (44.0)	139 (61.5)
		Once	72 (31.6)	Yes	135 (59.5)	183 (80.7)	158 (69.7)	123 (54.3)	95 (41.9)	135 (59.6)	127 (56.0)	87 (38.5)
		Regular	72 (31.8)									
		Mandatory	44 (19.2)									
Western Pacific		Never	0 (0.1)	No	99 (21.4)	164 (35.5)	159 (34.3)	145 (31.4)	195 (42.1)	45 (9.8)	117 (25.3)	193 (41.8)
		Once	45 (9.8)	Yes	363 (78.6)	298 (64.5)	303 (65.7)	317 (68.6)	267 (57.9)	417 (90.2)	345 (74.7)	269 (58.2)
		Regular	123 (26.7)									
		Mandatory	293 (63.5)									
**Income Level**												
Low-income	350	Never	15 (4.2)	No	209 (59.9)	178 (51.0)	245 (70.1)	231 (65.9)	269 (77.0)	45 (12.8)	275 (78.8)	237 (67.7)
		Once	113 (32.2)	Yes	140 (40.1)	171 (49.0)	105 (29.9)	119 (34.1)	80 (23.0)	305 (87.2)	74 (21.2)	113 (32.3)
		Regular	176 (50.3)									
		Mandatory	46 (13.3)									
Lower Middle-income	695	Never	28 (4)	No	293 (42.2)	197 (28.3)	277 (39.9)	158 (22.8)	328 (47.3)	118 (16.9)	325 (46.7)	465 (67.0)
		Once	176 (25.3)	Yes	402 (57.8)	498 (71.7)	418 (60.1)	536 (77.2)	366 (52.7)	577 (83.1)	370 (53.3)	230 (33.0)

	Nw		HH training HCWs		Training registry	WHO HH summary available	WHO Technical manual available	WHO HH brochure available	WHO glove use leaflet available	Active HH trainer	Training of HH observers	Budget for training
		Regular	239 (34.4)									
		Mandatory	252 (36.3)									
Upper Middle-income	1035	Never	45 (4.4)	No	333 (32.2)	239 (23.1)	251 (24.3)	244 (23.5)	300 (29.0)	208 (20.1)	374 (36.1)	591 (57.1)
		Once	128 (12.4)	Yes	702 (67.8)	796 (76.9)	784 (75.7)	791 (76.5)	735 (71.0)	827 (79.9)	661 (63.9)	444 (42.9)
		Regular	328 (31.7)									
		Mandatory	534 (51.6)									
High-income	1102	Never	3 (0.3)	No	203 (18.4)	213 (19.3)	239 (21.6)	235 (21.3)	332 (30.1)	50 (4.6)	253 (23.0)	526 (47.7)
		Once	109 (9.9)	Yes	900 (81.6)	889 (80.7)	864 (78.4)	867 (78.7)	770 (69.9)	1052 (95.4)	849 (77.0)	576 (52.3)
		Regular	291 (26.4)									
		Mandatory	699 (63.4)									
**Nationally coordinated**												
Yes	427	Never	15 (3.6)	No	150 (35.2)	159 (37.3)	134 (31.4)	148 (34.7)	176 (41.3)	37 (8.7)	201 (47.1)	313 (73.4)
		Once	86 (20.3)	Yes	276 (64.8)	268 (62.7)	293 (68.6)	279 (65.3)	250 (58.7)	389 (91.3)	226 (52.9)	114 (26.6)
		Regular	129 (30.2)									
		Mandatory	196 (46.0)									
No	2755	Never	75 (2.7)	No	887 (32.2)	668 (24.2)	878 (31.9)	720 (26.1)	1053 (38.2)	384 (13.9)	1027 (37.3)	1506 (54.7)
		Once	439 (15.9)	Yes	1868 (67.8)	2087 (75.8)	1878 (68.1)	2035 (73.9)	1702 (61.8)	2371 (86.1)	1729 (62.7)	1249 (45.3)
		Regular	905 (32.9)									

	Nw		HH training HCWs		Training registry	WHO HH summary available	WHO Technical manual available	WHO HH brochure available	WHO glove use leaflet available	Active HH trainer	Training of HH observers	Budget for training
		Mandatory	1335 (48.5)									
**Facility Type**												
Private	1528	Never	2 (0.1)	No	298 (19.5)	277 (18.1)	396 (25.9)	371 (24.3)	521 (34.1)	200 (13.1)	459 (30.0)	585 (38.3)
		Once	135 (8.8)	Yes	1230 (80.5)	1251 (81.9)	1132 (74.1)	1156 (75.7)	1007 (65.9)	1328 (86.9)	1069 (70.0)	943 (61.7)
		Regular	367 (24)									
		Mandatory	1024 (67)									
Public	1526	Never	88 (5.8)	No	699 (45.8)	529 (34.7)	585 (38.3)	465 (30.4)	665 (43.6)	215 (14.1)	715 (46.9)	1150 (75.4)
		Once	359 (23.5)	Yes	827 (54.2)	997 (65.3)	941 (61.7)	1061 (69.6)	861 (56.4)	1311 (85.9)	811 (53.1)	376 (24.6)
		Regular	596 (39.1)									
		Mandatory	483 (31.6)									
Other	128	Never	0 (0.3)	No	41 (31.9)	20 (15.7)	31 (24.1)	32 (24.9)	44 (34.5)	7 (5.3)	53 (41.5)	85 (66.3)
		Once	32 (25.1)	Yes	87 (68.1)	108 (84.3)	97 (75.9)	96 (75.1)	84 (65.5)	121 (94.7)	75 (58.5)	43 (33.7)
		Regular	71 (55.3)									
		Mandatory	25 (19.4)									
**Facility Level**												
Primary	2291	Never	81 (3.5)	No	701 (30.6)	640 (27.9)	730 (31.9)	688 (30.0)	863 (37.7)	306 (13.4)	848 (37.0)	1308 (57.1)
		Once	303 (13.2)	Yes	1590 (69.4)	1651 (72.1)	1560 (68.1)	1603 (70.0)	1427 (62.3)	1984 (86.6)	1442 (63.0)	983 (42.9)
		Regular	785 (34.3)									
		Mandatory	1122 (49)									
Secondary	416	Never	8 (1.9)	No	179 (43.0)	102 (24.6)	137 (32.9)	122 (29.3)	212 (51.0)	65 (15.7)	215 (51.7)	262 (63.0)

	Nw		HH training HCWs		Training registry	WHO HH summary available	WHO Technical manual available	WHO HH brochure available	WHO glove use leaflet available	Active HH trainer	Training of HH observers	Budget for training
		Once	114 (27.4)	Yes	237 (57.0)	314 (75.4)	279 (67.1)	294 (70.7)	204 (49.0)	351 (84.3)	201 (48.3)	154 (37.0)
		Regular	131 (31.5)									
		Mandatory	163 (39.2)									
Tertiary	318	Never	1 (0.4)	No	121 (38.0)	67 (21.0)	124 (38.9)	32 (10.2)	122 (38.2)	33 (10.3)	132 (41.5)	181 (56.8)
		Once	80 (25)	Yes	197 (62.0)	251 (79.0)	194 (61.1)	286 (89.8)	196 (61.8)	285 (89.7)	186 (58.5)	137 (43.2)
		Regular	79 (24.8)									
		Mandatory	158 (49.8)									
Other	157	Never	1 (0.6)	No	37 (23.5)	18 (11.5)	21 (13.1)	26 (16.5)	33 (20.9)	17 (10.5)	32 (20.3)	69 (44.0)
		Once	29 (18.5)	Yes	120 (76.5)	139 (88.5)	136 (86.9)	131 (83.5)	124 (79.1)	141 (89.5)	125 (79.7)	88 (56.0)
		Regular	39 (25.1)									
		Mandatory	88 (55.9)									

Nw, weighted frequency; WHO, World Health Organization; HH, hand hygiene; HCW, healthcare worker; IPC, infection prevention and control

Table S4.3a: Evaluation and Feedback: Weighted HHSAF responses specified per question
*Only includes surveys without missing values for element 3

	Nw		Ward-based audits	HH indication tested	HH technique tested	ABHR consumption monitored	Soap consumption monitored	ABHR 20L/1,000pd	Immediate feedback	Feedback to HCWs	Feedback to leadership
**Overall**	**3068**	**Response**	**Nw (%)**	**Nw (%)**	**Nw (%)**	**Nw (%)**	**Nw (%)**	**Nw (%)**	**Nw (%)**	**Nw (%)**	**Nw (%)**
		No	612 (20.0)	694 (22.6)	730 (23.8)	1168 (38.1)	1355 (44.1)	2108 (68.7)	902 (29.4)	1164 (37.9)	1096 (35.7)
		Yes	2456 (80.0)	2374 (77.4)	2338 (76.2)	1901 (61.9)	1714 (55.9)	960 (31.3)	2166 (70.6)	1904 (62.1)	1972 (64.3)
**WHO region**											
Africa	612	No	214 (34.9)	209 (34.1)	245 (40.0)	305 (49.9)	268 (43.8)	479 (78.2)	217 (35.4)	349 (57.0)	344 (56.2)
		Yes	399 (65.1)	404 (65.9)	368 (60.0)	307 (50.1)	344 (56.2)	133 (21.8)	395 (64.6)	263 (43.0)	268 (43.8)
Eastern Mediterranean	557	No	104 (18.7)	102 (18.3)	107 (19.2)	194 (34.8)	281 (50.5)	332 (59.6)	200 (35.9)	167 (30.0)	157 (28.1)
		Yes	453 (81.3)	455 (81.7)	450 (80.8)	363 (65.2)	275 (49.5)	225 (40.4)	357 (64.1)	390 (70.0)	400 (71.9)
Europe	628	No	84 (13.3)	132 (21.0)	139 (22.1)	192 (30.6)	293 (46.6)	374 (59.5)	174 (27.7)	279 (44.5)	227 (36.1)
		Yes	544 (86.7)	496 (79.0)	489 (77.9)	436 (69.4)	335 (53.4)	254 (40.5)	454 (72.3)	349 (55.5)	401 (63.9)
Americas	589	No	54 (9.2)	112 (19.1)	99 (16.8)	190 (32.2)	208 (35.3)	394 (66.8)	146 (24.8)	187 (31.7)	175 (29.7)
		Yes	535 (90.8)	477 (80.9)	491 (83.2)	400 (67.8)	381 (64.7)	196 (33.2)	443 (75.2)	402 (68.3)	415 (70.3)
South East Asia	227	No	78 (34.6)	63 (27.6)	49 (21.5)	91 (40.2)	71 (31.4)	168 (74.1)	77 (34.1)	105 (46.4)	115 (50.6)
		Yes	148 (65.4)	164 (72.4)	178 (78.5)	136 (59.8)	156 (68.6)	59 (25.9)	149 (65.9)	122 (53.6)	112 (49.4)
Western Pacific	455	No	78 (17.2)	77 (16.9)	92 (20.1)	195 (42.9)	233 (51.2)	362 (79.4)	88 (19.3)	77 (16.8)	79 (17.4)
		Yes	377 (82.8)	378 (83.1)	364 (79.9)	260 (57.1)	222 (48.8)	94 (20.6)	368 (80.7)	379 (83.2)	376 (82.6)
**Income Level**											
Low-income	331	No	162 (48.8)	148 (44.7)	186 (56.2)	175 (52.9)	188 (56.7)	303 (91.5)	150 (45.3)	205 (61.8)	219 (66.1)
		Yes	170 (51.2)	183 (55.3)	145 (43.8)	156 (47.1)	144 (43.3)	28 (8.5)	181 (54.7)	127 (38.2)	113 (33.9)

	Nw		Ward-based audits	HH indication tested	HH technique tested	ABHR consumption monitored	Soap consumption monitored	ABHR 20L/1,000pd	Immediate feedback	Feedback to HCWs	Feedback to leadership
Lower Middle-income	686	No	165 (24.0)	199 (29.1)	171 (24.9)	300 (43.8)	249 (36.3)	530 (77.2)	236 (34.3)	358 (52.1)	350 (51.0)
		Yes	522 (76.0)	487 (70.9)	515 (75.1)	386 (56.2)	437 (63.7)	157 (22.8)	451 (65.7)	328 (47.9)	336 (49.0)
Upper Middle-income	996	No	175 (17.6)	207 (20.8)	212 (21.3)	331 (33.2)	372 (37.3)	600 (60.3)	240 (24.1)	306 (30.7)	273 (27.4)
		Yes	821 (82.4)	789 (79.2)	784 (78.7)	666 (66.8)	624 (62.7)	396 (39.7)	756 (75.9)	690 (69.3)	723 (72.6)
High-income	1054	No	111 (10.5)	140 (13.2)	160 (15.2)	361 (34.3)	546 (51.8)	675 (64.0)	277 (26.2)	295 (28.0)	254 (24.1)
		Yes	944 (89.5)	915 (86.8)	894 (84.8)	693 (65.7)	509 (48.2)	380 (36.0)	778 (73.8)	759 (72.0)	801 (75.9)
**Nationally coordinated**											
Yes	426	No	117 (27.5)	121 (28.3)	134 (31.4)	183 (43)	182 (42.8)	310 (72.9)	167 (39.3)	160 (37.5)	154 (36.1)
		Yes	308 (72.5)	305 (71.7)	292 (68.6)	243 (57)	243 (57.2)	115 (27.1)	258 (60.7)	266 (62.5)	272 (63.9)
No	2643	No	495 (18.7)	574 (21.7)	596 (22.6)	985 (37.3)	1172 (44.4)	1798 (68)	735 (27.8)	1005 (38)	943 (35.7)
		Yes	2148 (81.3)	2069 (78.3)	2047 (77.4)	1658 (62.7)	1470 (55.6)	845 (32)	1908 (72.2)	1638 (62)	1700 (64.3)
**Facility Type**											
Private	1441	No	169 (11.7)	135 (9.4)	203 (14.1)	479 (33.2)	624 (43.3)	928 (64.4)	351 (24.4)	431 (29.9)	383 (26.5)
		Yes	1272 (88.3)	1306 (90.6)	1238 (85.9)	962 (66.8)	817 (56.7)	513 (35.6)	1090 (75.6)	1010 (70.1)	1059 (73.5)
Public	1500	No	428 (28.5)	537 (35.8)	515 (34.3)	654 (43.6)	691 (46.1)	1109 (73.9)	520 (34.7)	701 (46.7)	671 (44.7)
		Yes	1072 (71.5)	962 (64.2)	985 (65.7)	845 (56.4)	808 (53.9)	391 (26.1)	980 (65.3)	799 (53.3)	829 (55.3)
Other	128	No	16 (12.2)	22 (17.1)	12 (9.6)	35 (27.2)	39 (30.7)	71 (55.9)	31 (24.4)	33 (25.9)	43 (33.8)
		Yes	112 (87.8)	106 (82.9)	115 (90.4)	93 (72.8)	88 (69.3)	56 (44.1)	96 (75.6)	95 (74.1)	84 (66.2)

	Nw		Ward-based audits	HH indication tested	HH technique tested	ABHR consumption monitored	Soap consumption monitored	ABHR 20L/1,000pd	Immediate feedback	Feedback to HCWs	Feedback to leadership
**Facility Level**											
Primary	2182	No	394 (18.1)	432 (19.8)	462 (21.2)	811 (37.2)	890 (40.8)	1581 (72.5)	619 (28.3)	766 (35.1)	719 (33.0)
		Yes	1788 (81.9)	1749 (80.2)	1720 (78.8)	1371 (62.8)	1292 (59.2)	601 (27.5)	1563 (71.7)	1416 (64.9)	1463 (67.0)
Secondary	414	No	144 (34.8)	142 (34.4)	144 (34.8)	188 (45.3)	206 (49.6)	251 (60.7)	123 (29.7)	215 (52.0)	225 (54.2)
		Yes	270 (65.2)	272 (65.6)	270 (65.2)	227 (54.7)	209 (50.4)	163 (39.3)	291 (70.3)	199 (48.0)	190 (45.8)
Tertiary	315	No	56 (17.9)	104 (33.1)	108 (34.3)	114 (36.1)	177 (56.1)	175 (55.5)	111 (35.1)	144 (45.7)	127 (40.3)
		Yes	259 (82.1)	211 (66.9)	207 (65.7)	202 (63.9)	138 (43.9)	140 (44.5)	205 (64.9)	171 (54.3)	188 (59.7)
Other	157	No	17 (11.1)	15 (9.4)	16 (9.9)	55 (35.4)	82 (52.3)	101 (64.2)	50 (31.8)	39 (25.0)	25 (16.1)
		Yes	139 (88.9)	142 (90.6)	141 (90.1)	101 (64.6)	75 (47.7)	56 (35.8)	107 (68.2)	118 (75.0)	132 (83.9)

Nw, weighted frequency; HH,hand hygiene; ABHR, alcohol-based handrub; HCW, healthcare worker

Table S4.3b: Element 3 'Evaluation and Feedback': Weighted HHSAF responses specified per question
*Only includes surveys without missing values for element 3

	Nw	HH direct observation		HH compliance rate	
**Overall**	**3068**	**Response**	**Nw (%)**	**Response**	**Nw (%)**
		Never	538 (17.5)	<31%	618 (20.1)
		Irregular	502 (16.4)	31-40%	301 (9.8)
		Annual	650 (21.2)	41-50%	265 (8.6)
		Every 3m	1378 (44.9)	51-60%	311 (10.1)
				61-70%	414 (13.5)
				71-80%	418 (13.6)
				>80%	741 (24.1)
**WHO region**					
Africa	612	Never	161 (26.3)	<31%	181 (29.6)
		Irregular	143 (23.4)	31-40%	63 (10.3)
		Annual	24 (3.9)	41-50%	67 (10.9)
		Every 3m	284 (46.4)	51-60%	53 (8.7)
				61-70%	64 (10.5)
				71-80%	108 (17.6)
				>80%	76 (12.3)
Eastern Mediterranean	557	Never	86 (15.4)	<31%	98 (17.6)
		Irregular	55 (9.9)	31-40%	36 (6.5)
		Annual	149 (26.7)	41-50%	23 (4.1)
		Every 3m	267 (47.9)	51-60%	91 (16.3)
				61-70%	82 (14.8)
				71-80%	84 (15.0)
				>80%	143 (25.7)
Europe	628	Never	106 (16.9)	<31%	113 (18.0)
		Irregular	83 (13.2)	31-40%	67 (10.7)
		Annual	234 (37.2)	41-50%	21 (3.4)
		Every 3m	205 (32.7)	51-60%	64 (10.2)

	Nw	HH direct observation		HH compliance rate	
				61-70%	125 (20.0)
				71-80%	89 (14.2)
				>80%	148 (23.5)
Americas	589	Never	126 (21.3)	<31%	144 (24.4)
		Irregular	100 (16.9)	31-40%	94 (15.9)
		Annual	111 (18.8)	41-50%	51 (8.6)
		Every 3m	253 (42.9)	51-60%	36 (6.1)
				61-70%	86 (14.5)
				71-80%	40 (6.7)
				>80%	140 (23.8)
South East Asia	227	Never	47 (20.9)	<31%	46 (20.5)
		Irregular	69 (30.4)	31-40%	24 (10.7)
		Annual	64 (28.0)	41-50%	25 (11.1)
		Every 3m	47 (20.6)	51-60%	53 (23.4)
				61-70%	13 (5.7)
				71-80%	18 (8.1)
				>80%	47 (20.5)
Western Pacific	455	Never	12 (2.6)	<31%	35 (7.8)
		Irregular	52 (11.4)	31-40%	17 (3.7)
		Annual	70 (15.3)	41-50%	78 (17.1)
		Every 3m	322 (70.7)	51-60%	14 (3.1)
				61-70%	44 (9.7)
				71-80%	79 (17.4)
				>80%	188 (41.2)
**Income Level**					
Low-income	331	Never	99 (29.9)	<31%	120 (36.1)
		Irregular	102 (30.9)	31-40%	56 (16.8)
		Annual	8 (2.3)	41-50%	30 (8.9)

	Nw		HH direct observation		HH compliance rate
		Every 3m	122 (36.9)	51-60%	19 (5.9)
				61-70%	29 (8.7)
				71-80%	57 (17.1)
				>80%	22 (6.6)
Lower Middle-income	686	Never	186 (27.0)	<31%	199 (28.9)
		Irregular	173 (25.2)	31-40%	67 (9.8)
		Annual	80 (11.7)	41-50%	69 (10.0)
		Every 3m	248 (36.1)	51-60%	98 (14.2)
				61-70%	87 (12.6)
				71-80%	76 (11.0)
				>80%	92 (13.4)
Upper Middle-income	996	Never	140 (14.1)	<31%	155 (15.5)
		Irregular	108 (10.8)	31-40%	91 (9.1)
		Annual	266 (26.7)	41-50%	128 (12.9)
		Every 3m	482 (48.4)	51-60%	123 (12.3)
				61-70%	132 (13.3)
				71-80%	110 (11.1)
				>80%	258 (25.9)
High-income	1054	Never	113 (10.7)	<31%	145 (13.8)
		Irregular	119 (11.3)	31-40%	88 (8.3)
		Annual	297 (28.1)	41-50%	38 (3.6)
		Every 3m	525 (49.8)	51-60%	72 (6.8)
				61-70%	167 (15.8)
				71-80%	176 (16.7)
				>80%	369 (35.0)
**Nationally coordinated**					
Yes	426	Never	98 (23.1)	<31%	144 (33.8)
		Irregular	124 (29.2)	31-40%	62 (14.6)

	Nw		HH direct observation		HH compliance rate
		Annual	66<br>(15.5)	41-50%	32<br>(7.4)
		Every 3m	137<br>(32.1)	51-60%	22<br>(5.2)
				61-70%	68<br>(16.1)
				71-80%	33<br>(7.7)
				>80%	65<br>(15.2)
No	2643	Never	440<br>(16.6)	<31%	474<br>(18)
		Irregular	377<br>(14.3)	31-40%	239<br>(9.1)
		Annual	585<br>(22.1)	41-50%	233<br>(8.8)
		Every 3m	1241<br>(47)	51-60%	289<br>(10.9)
				61-70%	346<br>(13.1)
				71-80%	385<br>(14.6)
				>80%	676<br>(25.6)
**Facility Type**					
Private	1441	Never	139<br>(9.7)	<31%	176<br>(12.2)
		Irregular	139<br>(9.6)	31-40%	80<br>(5.5)
		Annual	328<br>(22.7)	41-50%	65<br>(4.5)
		Every 3m	836<br>(58.0)	51-60%	177<br>(12.3)
				61-70%	250<br>(17.4)
				71-80%	249<br>(17.2)
				>80%	444<br>(30.8)
Public	1500	Never	391<br>(26.1)	<31%	432<br>(28.8)
		Irregular	321<br>(21.4)	31-40%	198<br>(13.2)
		Annual	297<br>(19.8)	41-50%	189<br>(12.6)
		Every 3m	490<br>(32.6)	51-60%	118<br>(7.9)
				61-70%	148<br>(9.9)

	Nw	HH direct observation		HH compliance rate	
				71-80%	160 (10.7)
				>80%	253 (16.9)
Other	128	Never	8 (6.0)	<31%	10 (8.2)
		Irregular	42 (32.8)	31-40%	23 (18.1)
		Annual	25 (20.0)	41-50%	10 (7.8)
		Every 3m	53 (41.2)	51-60%	16 (12.4)
				61-70%	16 (12.3)
				71-80%	9 (7.4)
				>80%	43 (34.0)
**Facility Level**					
Primary	2182	Never	340 (15.6)	<31%	398 (18.2)
		Irregular	390 (17.9)	31-40%	255 (11.7)
		Annual	424 (19.4)	41-50%	161 (7.4)
		Every 3m	1028 (47.1)	51-60%	197 (9.0)
				61-70%	298 (13.6)
				71-80%	275 (12.6)
				>80%	598 (27.4)
Secondary	414	Never	90 (21.7)	<31%	103 (24.8)
		Irregular	66 (15.9)	31-40%	23 (5.6)
		Annual	132 (31.8)	41-50%	63 (15.2)
		Every 3m	127 (30.6)	51-60%	70 (16.8)
				61-70%	42 (10.0)
				71-80%	78 (18.9)
				>80%	36 (8.7)
Tertiary	315	Never	85 (26.9)	<31%	91 (28.9)
		Irregular	31 (9.8)	31-40%	15 (4.8)
		Annual	57 (17.9)	41-50%	34 (10.9)
		Every 3m	143 (45.4)	51-60%	34 (10.9)

	Nw	HH direct observation		HH compliance rate	
				61-70%	37 (11.7)
				71-80%	37 (11.6)
				>80%	67 (21.2)
Other	157	Never	24 (15.1)	<31%	27 (16.9)
		Irregular	15 (9.4)	31-40%	8 (5.0)
		Annual	39 (24.6)	41-50%	6 (4.0)
		Every 3m	80 (50.9)	51-60%	10 (6.5)
				61-70%	38 (24.4)
				71-80%	28 (18.1)
				>80%	39 (25.1)

Nw, weighted frequency; HH, hand hygiene; m, month

Table S4.4: Reminders in the Workplace: Weighted HHSAF responses specified per question
*Only includes surveys without missing values for element 4

	Nw		HH indication poster	Handrub poster	Handwashing poster		Poster audits		Other posters	HH leaflets at ward	Workplace reminders
**Overall**	**3117**	**Response**	**Nw (%)**	**Nw (%)**	**Nw (%)**	**Response**	**Nw (%)**	**Response**	**Nw (%)**	**Nw (%)**	**Nw (%)**
		No	264 (8.5)	374 (12)	211 (6.8)	Never	634 (20.3)	No	1170 (37.5)	1362 (43.7)	1713 (55.0)
		Some wards	439 (14.1)	457 (14.7)	429 (13.8)	Annual	1506 (48.3)	Yes	1947 (62.5)	1755 (56.3)	1404 (45.0)
		Most wards	645 (20.7)	608 (19.5)	707 (22.7)	2-3m	977 (31.3)				
		All wards	1770 (56.8)	1679 (53.9)	1770 (56.8)						
**WHO region**											
Africa	609	No	82 (13.5)	132 (21.8)	102 (16.8)	Never	180 (29.5)	No	290 (47.7)	319 (52.4)	395 (64.8)
		Some wards	100 (16.4)	121 (19.9)	99 (16.2)	Annual	190 (31.2)	Yes	318 (52.3)	290 (47.6)	214 (35.2)
		Most wards	122 (20.1)	75 (12.3)	166 (27.2)	2-3m	239 (39.3)				
		All wards	304 (50.0)	280 (46.0)	242 (39.8)						
Eastern Mediterranean	557	No	55 (9.8)	19 (3.4)	6 (1.1)	Never	53 (9.5)	No	112 (20.1)	159 (28.5)	201 (36.1)
		Some wards	44 (7.9)	44 (7.8)	56 (10.0)	Annual	249 (44.8)	Yes	445 (79.9)	398 (71.5)	356 (63.9)
		Most wards	96 (17.3)	120 (21.5)	109 (19.5)	2-3m	254 (45.6)				
		All wards	362 (65.1)	374 (67.3)	386 (69.4)						
Europe	629	No	21 (3.3)	24 (3.9)	6 (0.9)	Never	136 (21.6)	No	254 (40.3)	263 (41.9)	394 (62.7)
		Some wards	98 (15.6)	98 (15.6)	95 (15.1)	Annual	348 (55.3)	Yes	375 (59.7)	366 (58.1)	235 (37.3)
		Most wards	111 (17.6)	105 (16.8)	104 (16.5)	2-3m	145 (23.1)				
		All wards	399 (63.5)	401 (63.7)	425 (67.5)						
Americas	633	No	47 (7.5)	85 (13.4)	42 (6.7)	Never	107 (17.0)	No	263 (41.5)	324 (51.2)	389 (61.4)
		Some wards	128 (20.2)	143 (22.6)	144 (22.8)	Annual	316 (49.9)	Yes	370 (58.5)	309 (48.8)	244 (38.6)
		Most wards	195 (30.8)	191 (30.1)	186 (29.3)	2-3m	209 (33.1)				
		All wards	263 (41.5)	215 (33.9)	261 (41.2)						
South East Asia	227	No	48 (21.1)	92 (40.8)	38 (16.9)	Never	91 (39.9)	No	104 (45.9)	111 (48.9)	113 (49.9)
		Some wards	44 (19.4)	32 (14.2)	20 (8.8)	Annual	112 (49.4)	Yes	123 (54.1)	116 (51.1)	114 (50.1)
		Most wards	31 (13.8)	25 (11)	53 (23.3)	2-3m	24 (10.7)				
		All wards	104 (45.7)	77 (34)	116 (51)						
Western Pacific	463	No	11 (2.4)	21 (4.4)	17 (3.6)	Never	68 (14.6)	No	148 (31.8)	186 (40.2)	222 (47.9)
		Some wards	25 (5.4)	19 (4.0)	15 (3.3)	Annual	290 (62.7)	Yes	316 (68.2)	277 (59.8)	242 (52.1)

	Nw		HH indication poster	Handrub poster	Handwashing poster		Poster audits		Other posters	HH leaflets at ward	Workplace reminders
		Most wards	89 (19.3)	93 (20.0)	90 (19.5)	2-3m	105 (22.7)				
		All wards	338 (72.9)	332 (71.6)	341 (73.6)						
**Income Level**											
Low-income	318	No	53 (16.6)	91 (28.5)	77 (24.3)	Never	103 (32.5)	No	149 (46.9)	221 (69.6)	262 (82.4)
		Some wards	89 (27.9)	99 (31.1)	82 (25.9)	Annual	134 (42.0)	Yes	169 (53.1)	97 (30.4)	56 (17.6)
		Most wards	92 (29.0)	43 (13.5)	131 (41.1)	2-3m	81 (25.5)				
		All wards	84 (26.5)	86 (27.0)	28 (8.7)						
Lower Middle-income	697	No	117 (16.7)	155 (22.2)	75 (10.8)	Never	201 (28.9)	No	306 (44)	354 (50.7)	397 (57)
		Some wards	114 (16.3)	105 (15.0)	108 (15.4)	Annual	311 (44.7)	Yes	390 (56)	343 (49.3)	300 (43)
		Most wards	148 (21.3)	194 (27.8)	219 (31.5)	2-3m	184 (26.4)				
		All wards	318 (45.6)	244 (35.0)	294 (42.3)						
Upper Middle-income	1032	No	56 (5.4)	64 (6.2)	37 (3.6)	Never	136 (13.2)	No	389 (37.7)	447 (43.3)	509 (49.3)
		Some wards	113 (11.0)	108 (10.4)	90 (8.7)	Annual	497 (48.2)	Yes	643 (62.3)	585 (56.7)	523 (50.7)
		Most wards	175 (16.9)	187 (18.1)	149 (14.5)	2-3m	398 (38.6)				
		All wards	688 (66.7)	673 (65.2)	755 (73.2)						
High-income	1071	No	39 (3.6)	64 (6.0)	21 (2.0)	Never	193 (18.0)	No	325 (30.4)	341 (31.8)	545 (50.9)
		Some wards	123 (11.5)	146 (13.6)	149 (13.9)	Annual	564 (52.6)	Yes	745 (69.6)	730 (68.2)	526 (49.1)
		Most wards	230 (21.4)	185 (17.2)	207 (19.4)	2-3m	314 (29.3)				
		All wards	679 (63.4)	676 (63.2)	693 (64.7)						
**Nationally coordinated**											
Yes	427	No	15 (3.5)	44 (10.2)	20 (4.6)	Never	82 (19.3)	No	204 (47.8)	227 (53.3)	261 (61.3)
		Some wards	96 (22.4)	110 (25.8)	72 (16.9)	Annual	259 (60.8)	Yes	223 (52.2)	199 (46.7)	165 (38.7)
		Most wards	103 (24.1)	55 (13.0)	133 (31.1)	2-3m	85 (20.0)				
		All wards	213 (50.0)	218 (51.0)	202 (47.4)						
No	2690	No	249 (9.3)	330 (12.3)	191 (7.1)	Never	552 (20.5)	No	966 (35.9)	1135 (42.2)	1452 (54.0)
		Some wards	343 (12.8)	347 (12.9)	357 (13.3)	Annual	1247 (46.3)	Yes	1724 (64.1)	1556 (57.8)	1239 (46.0)
		Most wards	542 (20.1)	552 (20.5)	574 (21.3)	2-3m	892 (33.1)				
		All wards	1556 (57.8)	1461 (54.3)	1568 (58.3)						
**Facility Type**											

	Nw		HH indication poster	Handrub poster	Handwashing poster		Poster audits		Other posters	HH leaflets at ward	Workplace reminders
Private	1480	No	58 (3.9)	148 (10.0)	96 (6.5)	Never	182 (12.3)	No	417 (28.2)	506 (34.2)	683 (46.1)
		Some wards	123 (8.3)	116 (7.8)	100 (6.7)	Annual	679 (45.9)	Yes	1063 (71.8)	974 (65.8)	797 (53.9)
		Most wards	227 (15.3)	220 (14.9)	241 (16.3)	2-3m	618 (41.8)				
		All wards	1072 (72.4)	995 (67.2)	1043 (70.5)						
Public	1509	No	200 (13.2)	219 (14.5)	115 (7.6)	Never	440 (29.2)	No	717 (47.5)	803 (53.2)	962 (63.7)
		Some wards	282 (18.7)	304 (20.1)	294 (19.5)	Annual	766 (50.8)	Yes	792 (52.5)	706 (46.8)	548 (36.3)
		Most wards	409 (27.1)	375 (24.9)	451 (29.9)	2-3m	303 (20.1)				
		All wards	619 (41.0)	611 (40.5)	649 (43)						
Other	128	No	6 (4.7)	6 (4.6)	0 (0.2)	Never	12 (9.2)	No	36 (28.2)	53 (41.3)	69 (53.7)
		Some wards	33 (26.1)	37 (29.3)	35 (27.5)	Annual	60 (47.3)	Yes	92 (71.8)	75 (58.7)	59 (46.3)
		Most wards	10 (7.5)	13 (9.8)	14 (11.1)	2-3m	56 (43.5)				
		All wards	79 (61.7)	72 (56.4)	78 (61.1)						
**Facility Level**											
Primary	2230	No	196 (8.8)	309 (13.8)	177 (7.9)	Never	453 (20.3)	No	835 (37.4)	890 (39.9)	1241 (55.6)
		Some wards	307 (13.7)	331 (14.8)	299 (13.4)	Annual	1074 (48.1)	Yes	1396 (62.6)	1341 (60.1)	990 (44.4)
		Most wards	436 (19.5)	385 (17.2)	460 (20.6)	2-3m	703 (31.5)				
		All wards	1292 (57.9)	1206 (54.1)	1294 (58)						
Secondary	414	No	25 (5.9)	43 (10.3)	17 (4.1)	Never	104 (25.1)	No	189 (45.6)	261 (63.1)	233 (56.3)
		Some wards	81 (19.5)	84 (20.2)	86 (20.7)	Annual	206 (49.7)	Yes	225 (54.4)	153 (36.9)	181 (43.7)
		Most wards	117 (28.3)	105 (25.3)	127 (30.6)	2-3m	104 (25.2)				
		All wards	192 (46.3)	183 (44.2)	185 (44.7)						
Tertiary	316	No	36 (11.4)	17 (5.5)	12 (3.7)	Never	62 (19.8)	No	109 (34.5)	164 (51.9)	169 (53.4)
		Some wards	37 (11.8)	27 (8.7)	26 (8.4)	Annual	147 (46.6)	Yes	207 (65.5)	152 (48.1)	147 (46.6)
		Most wards	65 (20.7)	92 (29.2)	97 (30.8)	2-3m	106 (33.7)				
		All wards	177 (56.1)	179 (56.6)	180 (57.2)						
Other	157	No	7 (4.7)	5 (3.3)	5 (3.4)	Never	15 (9.3)	No	38 (23.9)	47 (30.2)	70 (44.9)
		Some wards	14 (9.1)	15 (9.5)	18 (11.3)	Annual	79 (50.5)	Yes	119 (76.1)	110 (69.8)	86 (55.1)
		Most wards	26 (16.7)	26 (16.8)	23 (14.8)	2-3m	63 (40.2)				
		All wards	109 (69.5)	110 (70.4)	111 (70.5)						

Nw, weighted frequency; HH, hand hygiene; IPC, infection prevention and control

Table S4.5a: Institutional Safety Climate (Indicator 1-4): Weighted HHSAF responses specified per question
*Only includes surveys without missing values for element 5

	Nw		HH team (0/5)	HH team meetings	HH team dedi-cated budget	CEO commitment)	Med. director commitment	Nurse director commitment	HH May 5th plan	HH champions	HH Role models
**Overall**	**3059**	**Response**	**Nw (%)**	**Nw (%)**	**Nw (%)**	**Nw (%)**	**Nw (%)**	**Nw (%)**	**Nw (%)**	**Nw (%)**	**Nw (%)**
		No	1160 (37.9)	1677 (54.8)	1629 (53.3)	768 (25.1)	670 (21.9)	429 (14.0)	1134 (37.1)	1863 (60.9)	1948 (63.7)
		Yes	1899 (62.1)	1382 (45.2)	1430 (46.7)	2291 (74.9)	2389 (78.1)	2630 (86.0)	1925 (62.9)	1196 (39.1)	1111 (36.3)
**WHO region**											
Africa	593	No	179 (30.2)	305 (51.4)	331 (55.8)	83 (14.0)	76 (12.9)	64 (10.7)	359 (60.5)	335 (56.5)	395 (66.6)
		Yes	414 (69.8)	288 (48.6)	262 (44.2)	510 (86.0)	517 (87.1)	530 (89.3)	235 (39.5)	258 (43.5)	198 (33.4)
Eastern Mediterranean	556	No	231 (41.6)	291 (52.2)	292 (52.4)	81 (14.6)	89 (16.1)	64 (11.5)	120 (21.6)	349 (62.7)	364 (65.4)
		Yes	325 (58.4)	266 (47.8)	265 (47.6)	475 (85.4)	467 (83.9)	492 (88.5)	436 (78.4)	207 (37.3)	192 (34.6)
Europe	629	No	179 (28.4)	283 (45.1)	267 (42.4)	209 (33.2)	173 (27.5)	78 (12.5)	204 (32.5)	437 (69.5)	394 (62.6)
		Yes	450 (71.6)	345 (54.9)	362 (57.6)	420 (66.8)	456 (72.5)	550 (87.5)	425 (67.5)	192 (30.5)	235 (37.4)
Americas	613	No	226 (36.9)	338 (55.2)	361 (59.0)	226 (36.9)	175 (28.5)	101 (16.5)	205 (33.5)	372 (60.8)	392 (63.9)
		Yes	386 (63.1)	275 (44.8)	251 (41.0)	387 (63.1)	438 (71.5)	511 (83.5)	408 (66.5)	240 (39.2)	221 (36.1)
South East Asia	223	No	141 (63.0)	160 (71.7)	145 (64.8)	92 (41.1)	72 (32.2)	59 (26.6)	142 (63.4)	155 (69.5)	152 (67.9)
		Yes	83 (37.0)	63 (28.3)	79 (35.2)	131 (58.9)	151 (67.8)	164 (73.4)	82 (36.6)	68 (30.5)	72 (32.1)
Western Pacific	445	No	204 (45.9)	300 (67.5)	234 (52.6)	78 (17.5)	85 (19.0)	62 (14.0)	105 (23.5)	214 (48.1)	252 (56.6)
		Yes	241 (54.1)	145 (32.5)	211 (47.4)	367 (82.5)	360 (81.0)	382 (86.0)	340 (76.5)	231 (51.9)	193 (43.4)
**Income Level**											
Low-income	314	No	122 (38.9)	172 (54.6)	196 (62.3)	73 (23.2)	57 (18.2)	51 (16.4)	209 (66.4)	252 (80.2)	288 (91.6)
		Yes	192 (61.1)	143 (45.4)	118 (37.7)	241 (76.8)	257 (81.8)	263 (83.6)	106 (33.6)	62 (19.8)	26 (8.4)
Lower Middle-income	683	No	266 (38.9)	422 (61.8)	397 (58.2)	207 (30.2)	130 (19.0)	130 (19.1)	370 (54.1)	430 (63.0)	436 (63.9)
		Yes	417 (61.1)	261 (38.2)	285 (41.8)	476 (69.8)	553 (81.0)	553 (80.9)	313 (45.9)	253 (37.0)	247 (36.1)
Upper Middle-income	1024	No	414 (40.4)	547 (53.4)	518 (50.6)	286 (28.0)	270 (26.3)	131 (12.8)	277 (27)	632 (61.7)	617 (60.3)
		Yes	610 (59.6)	477 (46.6)	506 (49.4)	738 (72.0)	755 (73.7)	893 (87.2)	748 (73.0)	392 (38.3)	407 (39.7)
High-income	1038	No	359 (34.5)	537 (51.7)	518 (49.9)	203 (19.5)	214 (20.6)	116 (11.2)	279 (26.9)	549 (52.9)	607 (58.5)
		Yes	679 (65.5)	501 (48.3)	520 (50.1)	835 (80.5)	824 (79.4)	922 (88.8)	758 (73.1)	489 (47.1)	431 (41.5)
**Nationally coordinated**											

	Nw		HH team (0/5)	HH team meetings	HH team dedi-cated budget	CEO commitment)	Med. director commitment	Nurse director commitment	HH May 5th plan	HH champions	HH Role models
Yes	408	No	207 (50.8)	262 (64.2)	263 (64.6)	133 (32.6)	143 (35.1)	96 (23.6)	205 (50.2)	265 (65.0)	300 (73.5)
		Yes	201 (49.2)	146 (35.8)	144 (35.4)	275 (67.4)	264 (64.9)	311 (76.4)	203 (49.8)	143 (35.0)	108 (26.5)
No	2651	No	953 (36.0)	1416 (53.4)	1366 (51.5)	636 (24.0)	527 (19.9)	332 (12.5)	930 (35.1)	1598 (60.3)	1648 (62.2)
		Yes	1698 (64.0)	1236 (46.6)	1285 (48.5)	2016 (76.0)	2124 (80.1)	2319 (87.5)	1722 (64.9)	1053 (39.7)	1003 (37.8)
**Facility Type**											
Private	1456	No	407 (28.0)	643 (44.1)	643 (44.1)	238 (16.3)	222 (15.3)	150 (10.3)	417 (28.6)	736 (50.5)	794 (54.5)
		Yes	1049 (72.0)	814 (55.9)	814 (55.9)	1219 (83.7)	1234 (84.7)	1307 (89.7)	1040 (71.4)	721 (49.5)	663 (45.5)
Public	1475	No	729 (49.4)	986 (66.9)	931 (63.2)	490 (33.2)	425 (28.8)	264 (17.9)	679 (46.0)	1057 (71.6)	1087 (73.7)
		Yes	746 (50.6)	489 (33.1)	544 (36.8)	985 (66.8)	1050 (71.2)	1211 (82.1)	796 (54.0)	418 (28.4)	388 (26.3)
Other	128	No	24 (19.0)	48 (37.8)	55 (43.1)	41 (32.1)	23 (18.4)	15 (11.6)	39 (30.4)	71 (55.6)	67 (52.6)
		Yes	103 (81.0)	79 (62.2)	73 (56.9)	87 (67.9)	104 (81.6)	113 (88.4)	89 (69.6)	57 (44.4)	61 (47.4)
**Facility Level**											
Primary	2182	No	790 (36.2)	1196 (54.8)	1163 (53.3)	604 (27.7)	496 (22.8)	313 (14.3)	833 (38.2)	1243 (57.0)	1306 (59.9)
		Yes	1392 (63.8)	985 (45.2)	1018 (46.7)	1578 (72.3)	1685 (77.2)	1869 (85.7)	1348 (61.8)	938 (43.0)	876 (40.1)
Secondary	409	No	200 (48.8)	241 (58.9)	250 (61.1)	108 (26.5)	76 (18.6)	58 (14.2)	147 (35.8)	307 (75.0)	317 (77.3)
		Yes	209 (51.2)	168 (41.1)	159 (38.9)	301 (73.5)	333 (81.4)	351 (85.8)	263 (64.2)	102 (25.0)	93 (22.7)
Tertiary	314	No	108 (34.5)	156 (49.7)	130 (41.4)	41 (13.0)	85 (27.1)	52 (16.5)	119 (37.9)	227 (72.2)	227 (72.3)
		Yes	206 (65.5)	158 (50.3)	184 (58.6)	273 (87.0)	229 (72.9)	262 (83.5)	195 (62.1)	87 (27.8)	87 (27.7)
Other	154	No	62 (40.6)	83 (54.3)	86 (55.8)	15 (9.8)	12 (8.0)	6 (4.0)	35 (22.7)	86 (55.9)	98 (63.8)
		Yes	91 (59.4)	70 (45.7)	68 (44.2)	139 (90.2)	141 (92.0)	147 (96.0)	119 (77.3)	68 (44.1)	56 (36.2)

Nw, weighted frequency; CEO, chief executive officer; HH, hand hygiene; IPC, infection prevention and control

Table S4.5b: Institutional Safety Climate (Indicator 5 -6): Weighted HHSAF responses specified per question
*Only includes surveys without missing values for element 5

	Nw		HH patient leaflet	HH patient engangement	HH e-learning	HH target institute	Sharing inno-vations	HH communi-cation	Personal ac-countability	Buddy system
**Overall**	**3059**	**Response**	**Nw (%)**	**Nw (%)**	**Nw (%)**	**Nw (%)**	**Nw (%)**	**Nw (%)**	**Nw (%)**	**Nw (%)**
		No	1132 (37.0)	2143 (70.0)	1651 (54.0)	1263 (41.3)	1789 (58.5)	1131 (37)	1590 (52.0)	1326 (43.3)
		Yes	1927 (63.0)	916 (30.0)	1408 (46.0)	1796 (58.7)	1270 (41.5)	1928 (63.0)	1469 (4.08)	1733 (56.7)
**Region**										
Africa	593	No	303 (51.1)	473 (79.7)	434 (73.2)	411 (69.2)	367 (61.9)	204 (34.5)	248 (41.9)	241 (40.6)
		Yes	290 (48.9)	120 (20.3)	159 (26.8)	182 (30.8)	226 (38.1)	389 (65.5)	345 (58.1)	352 (59.4)
Eastern mediterranean	556	No	188 (33.9)	334 (60.0)	274 (49.3)	164 (29.5)	285 (51.2)	228 (40.9)	260 (46.8)	213 (38.3)
		Yes	368 (66.1)	223 (40.0)	282 (50.7)	392 (70.5)	271 (48.8)	329 (59.1)	296 (53.2)	343 (61.7)
Europe	629	No	196 (31.2)	462 (73.5)	339 (53.9)	250 (39.7)	378 (60.1)	274 (43.5)	367 (58.4)	261 (41.6)
		Yes	433 (68.8)	167 (26.5)	290 (46.1)	379 (60.3)	251 (39.9)	355 (56.5)	262 (41.6)	367 (58.4)
Americas	613	No	234 (38.2)	410 (67.0)	188 (30.6)	230 (37.6)	381 (62.2)	228 (37.2)	342 (55.8)	303 (49.4)
		Yes	378 (61.8)	202 (33.0)	425 (69.4)	382 (62.4)	231 (37.8)	385 (62.8)	271 (44.2)	310 (50.6)
South East Asia	223	No	113 (50.7)	155 (69.2)	175 (78.4)	126 (56.6)	146 (65.4)	133 (59.7)	119 (53.4)	118 (53.0)
		Yes	110 (49.3)	69 (30.8)	48 (21.6)	97 (43.4)	77 (34.6)	90 (40.3)	104 (46.6)	105 (47.0)
Western Pacific	445	No	97 (21.9)	310 (69.6)	241 (54.2)	82 (18.5)	232 (52.2)	64 (14.4)	253 (56.8)	189 (42.6)
		Yes	347 (78.1)	135 (30.4)	204 (45.8)	363 (81.5)	213 (47.8)	381 (85.6)	192 (43.2)	256 (57.4)
**Income Level**										
Low-income	314	No	198 (63.1)	271 (86.1)	251 (80.0)	264 (84.1)	262 (83.4)	142 (45.2)	148 (47.1)	143 (45.6)
		Yes	116 (36.9)	44 (13.9)	63 (20.0)	50 (15.9)	52 (16.6)	172 (54.8)	166 (52.9)	171 (54.4)
Lower Middle-income	683	No	360 (52.7)	549 (80.4)	463 (67.8)	382 (55.9)	448 (65.6)	336 (49.2)	361 (52.8)	354 (51.9)
		Yes	323 (47.3)	134 (19.6)	220 (32.2)	301 (44.1)	235 (34.4)	347 (50.8)	322 (47.2)	329 (48.1)
Upper Middle-income	1024	No	289 (28.2)	633 (61.8)	573 (55.9)	310 (30.3)	561 (54.8)	350 (34.2)	547 (53.4)	424 (41.4)
		Yes	735 (71.8)	391 (38.2)	451 (44.1)	714 (69.7)	463 (45.2)	674 (65.8)	477 (46.6)	600 (58.6)
High-income	1038	No	285 (27.5)	690 (66.5)	364 (35.0)	307 (29.6)	518 (49.9)	303 (29.2)	534 (51.5)	404 (38.9)
		Yes	752 (72.5)	348 (33.5)	674 (65)	731 (70.4)	519 (50.1)	735 (70.8)	503 (48.5)	634 (61.1)
**Nationally coordinated**										
Yes	408	No	191 (46.8)	313 (76.8)	249 (61.1)	201 (49.3)	263 (64.4)	208 (51.0)	253 (62.1)	203 (49.7)

	Nw		HH patient leaflet	HH patient engangement	HH e-learning	HH target institute	Sharing inno-vations	HH communi-cation	Personal ac-countability	Buddy system
		Yes	217 (53.2)	95 (23.2)	159 (38.9)	207 (50.7)	145 (35.6)	200 (49.0)	155 (37.9)	205 (50.3)
No	2651	No	942 (35.5)	1830 (69.0)	1402 (52.9)	1062 (40.1)	1527 (57.6)	923 (34.8)	1337 (50.4)	1123 (42.4)
		Yes	1710 (64.5)	822 (31.0)	1249 (47.1)	1589 (59.9)	1125 (42.4)	1728 (65.2)	1314 (49.6)	1528 (57.6)
**Facility Type**										
Private	1456	No	368 (25.2)	939 (64.5)	710 (48.8)	454 (31.2)	692 (47.5)	441 (30.3)	553 (38.0)	469 (32.2)
		Yes	1089 (74.8)	517 (35.5)	746 (51.2)	1002 (68.8)	764 (52.5)	1015 (69.7)	903 (62.0)	987 (67.8)
Public	1475	No	686 (46.5)	1093 (74.1)	874 (59.3)	757 (51.3)	1019 (69.1)	637 (43.2)	989 (67.1)	833 (56.5)
		Yes	789 (53.5)	382 (25.9)	601 (40.7)	718 (48.7)	456 (30.9)	838 (56.8)	486 (32.9)	641 (43.5)
Other	128	No	78 (61.3)	110 (86.6)	67 (52.2)	52 (40.4)	78 (60.9)	53 (41.4)	47 (37.0)	23 (18.1)
		Yes	49 (38.7)	17 (13.4)	61 (47.8)	76 (59.6)	50 (39.1)	75 (58.6)	80 (63.0)	104 (81.9)
**Facility Level**										
Primary	2182	No	759 (34.8)	1545 (70.8)	1157 (53.0)	909 (41.7)	1247 (57.2)	785 (36.0)	1072 (49.2)	879 (40.3)
		Yes	1422 (65.2)	637 (29.2)	1025 (47.0)	1273 (58.3)	935 (42.8)	1397 (64.0)	1109 (50.8)	1302 (59.7)
Secondary	409	No	205 (50.0)	292 (71.3)	249 (60.8)	183 (44.6)	299 (73.0)	185 (45.3)	266 (65.1)	228 (55.6)
		Yes	205 (50.0)	117 (28.7)	160 (39.2)	227 (55.4)	111 (27.0)	224 (54.7)	143 (34.9)	182 (44.4)
Tertiary	314	No	139 (44.1)	222 (70.7)	184 (58.5)	127 (40.3)	160 (50.8)	118 (37.6)	190 (60.4)	172 (54.9)
		Yes	176 (55.9)	92 (29.3)	130 (41.5)	187 (59.7)	154 (49.2)	196 (62.4)	124 (39.6)	142 (45.1)
Other	154	No	30 (19.4)	83 (54.3)	62 (40.1)	45 (29.0)	84 (54.4)	43 (27.7)	61 (39.8)	46 (30.1)
		Yes	124 (80.6)	70 (45.7)	92 (59.9)	109 (71.0)	70 (45.6)	111 (72.3)	92 (60.2)	107 (69.9)

Nw, weighted frequency; HH, hand hygiene; IPC, infection prevention and control

Supplementary File S5: Differences in element-specific scores for the Hand Hygiene Self-Assessment Framework (HHSAF) survey 2019 versus 2015

Only includes survey responses from healthcare facilities that responded to both surveys (N=192)

**Element**		**HHSAF Survey 2015**	**HHSAF Survey 2019**	**Median score difference 2019-2015**	**Comparison**
	**N**	**Median (IQR)**	**Median (IQR)**	**Median of score differences# (IQR)**	**P-value***
System change	192	100 (85-100)	95 (83.8-100)	0 (-10-0)	0.18
Training and Education	188	90 (75-100)	90 (75-100)	0 (-10-10)	0.84
Evaluation and Feedback	185	90 (75-95)	90 (80-95)	0 (-5-10)	0.090
Reminders in the Workplace	189	90 (72.5-95)	85 (75-95)	0 (-10-10)	0.77
Institutional Safety Climate	188	75 (55-90)	75 (65-90)	5 (-6.2-15)	**0.0027**
HH Leadership	114	15 (14-18)	16 (14-18)	0 (-2-2)	0.71

IQR, Interquartile range; HH, Hand Hygiene

This takes into account the pairing of survey responses, whereby the median of the difference will not be equal to subtraction of the overall median scores for 2019 and 2015

* Wilcoxon signed-rank test for paired data
